# The Role of Land Use Types and Water Chemical Properties in Structuring the Microbiomes of a Connected Lake System

**DOI:** 10.3389/fmicb.2020.00089

**Published:** 2020-02-12

**Authors:** Sophi Marmen, Lior Blank, Ashraf Al-Ashhab, Assaf Malik, Lars Ganzert, Maya Lalzar, Hans-Peter Grossart, Daniel Sher

**Affiliations:** ^1^Department of Marine Biology, Leon H. Charney School of Marine Sciences, University of Haifa, Haifa, Israel; ^2^Department of Plant Pathology and Weed Research, ARO, Volcani Center, Rishon Lezion, Israel; ^3^Microbial Metagenomics Division, Dead Sea and Arava Science Center, Masada, Israel; ^4^Bioinformatics Service Unit, University of Haifa, Haifa, Israel; ^5^Department of Experimental Limnology, Leibniz Institute of Freshwater Ecology and Inland Fisheries, Stechlin, Germany; ^6^Institute of Biochemistry and Biology, University of Potsdam, Potsdam, Germany

**Keywords:** microbiome, lakes, water parameters, land use, distribution, cyanobacteria

## Abstract

Lakes and other freshwater bodies are intimately connected to the surrounding land, yet to what extent land-use affects the quality of freshwater and the microbial communities living in various freshwater environments is largely unknown. We address this question through an analysis of the land use surrounding 46 inter-connected lakes located within seven different drainage basins in northern Germany, and the microbiomes of these lakes during early summer. Lake microbiome structure was not correlated with the specific drainage basin or by basin size, and bacterial distribution did not seem to be limited by distance. Instead, land use within the drainage basin could predict, to some extent, NO_2_ + NO_3_ concentrations in the water, which (together with temperature, *chlorophyll a* and total phosphorus) correlated to some extent with the water microbiome structure. Land use directly surrounding the water bodies, however, had little observable effects on water quality or the microbiome. Several microbial lineages, including Cyanobacteria and Verrucomicrobia, were differentially partitioned between the lakes. Significantly more data, including time-series measurements of land use and water chemical properties, are needed to fully understand the interaction between the environment and the organization of microbial communities.

## Introduction

The world’s population growth is maintained by a suite of human enterprises such as agriculture, industry, fishing, and international commerce (Vitousek et al., 1997). The use of land for such enterprises has greatly altered the way ecosystems interact with the surrounding land, atmosphere and aquatic systems (Vitousek et al., 1997). Freshwater ecosystems, in particular, may be highly sensitive to human impact, mainly through eutrophication.

The microbial communities (“microbiomes”) living in freshwater ecosystems are intimately connected with water quality. Microbes take up, utilize and recycle elements such as carbon, nitrogen, phosphorus and sulfur, and thus control and modulate local and global elemental cycles (Newton et al., 2011). Some aquatic microorganisms are potential pathogens of animals or humans, or can produce secondary metabolites or toxins (Abdelzaher et al., 2010; Cabral, 2010; Valério et al., 2010). Thus, significant effort has been invested in elucidating the environmental drivers that determine the composition and function of freshwater microbiomes (reviewed by e.g., Logue and Lindström, 2008; Williamson et al., 2009; Newton et al., 2011; Zeglin, 2015). Environmental drivers of microbial community structure may act at the local scale, where the physical–chemical characteristics of the water lead to a “species sorting” process which enrich for bacteria adapted to occupy that specific niche (Jones and McMahon, 2009; Langenheder and Székely, 2011; Van Rossum et al., 2015). Such physical - chemical properties can include, for example, nutrient, organic carbon or metal concentrations, pH, temperature, salinity and light intensity (Crump et al., 2004; Horner-Devine et al., 2004; Yannarell and Triplett, 2005; Zeglin, 2015). The physical-chemical properties of water depend, in turn, at least partly on the input of nutrients, organic matter and other contaminants from the surrounding terrestrial ecosystem via the watershed (Chen et al., 2018). Specifically, different types of land use surrounding the water bodies (e.g., forests, agricultural lands, urban lands, etc.) can strongly affect the quantity and quality of terrestrial input into water bodies (reviewed by Solomon et al., 2015).

Local processes can be affected by processes occurring at larger spatial and temporal scales. For example, physical connectivity between water bodies (e.g., within the same drainage basin) may lead to bacterial transfer between environments, a process termed “mass effect’,’ (e.g., Quinn et al., 1997; Nelson et al., 2009; Crump et al., 2012; Lindström and Langenheder, 2012; Logue et al., 2012). In very strongly connected habitats, dispersal rates may be so high that they lead to ecosystem homogenization (Leibold and Norberg, 2004). As the spatial scales increase, geographic distances and physical barriers may lead to limitations on bacterial dispersal, resulting in “distance decay” patterns, whereby differences between microbial communities increase with distance (Van der Gucht et al., 2007; Lear et al., 2013; Niño-García et al., 2016; Marcelino et al., 2017; Logares et al., 2018). As the various processes affecting freshwater microbial ecosystems are identified and characterized, many questions remain: to what extent are local conditions within water bodies affected by those in the surrounding terrestrial system? What is the relative importance of local conditions in determining microbial community structure? What is the role of mass transport of microbes from upstream sites, and how does this differ between freshwater systems? Are all microbial taxa affected in the same manner by these processes? While many of these questions have been addressed in ecosystems ranging from highly impacted agricultural lands (e.g., Griffin et al., 2017) to pristine Antarctic lakes (Logares et al., 2018), few studies have aimed to integrate the effects of hydrology and water chemistry on lake biology in the context of land-use within their respective catchment basins (e.g., Catherine et al., 2016). Answering some of these questions is critical as we aim to understand how changes in the environment, including land use, affect aquatic ecosystems.

**FIGURE 1:**
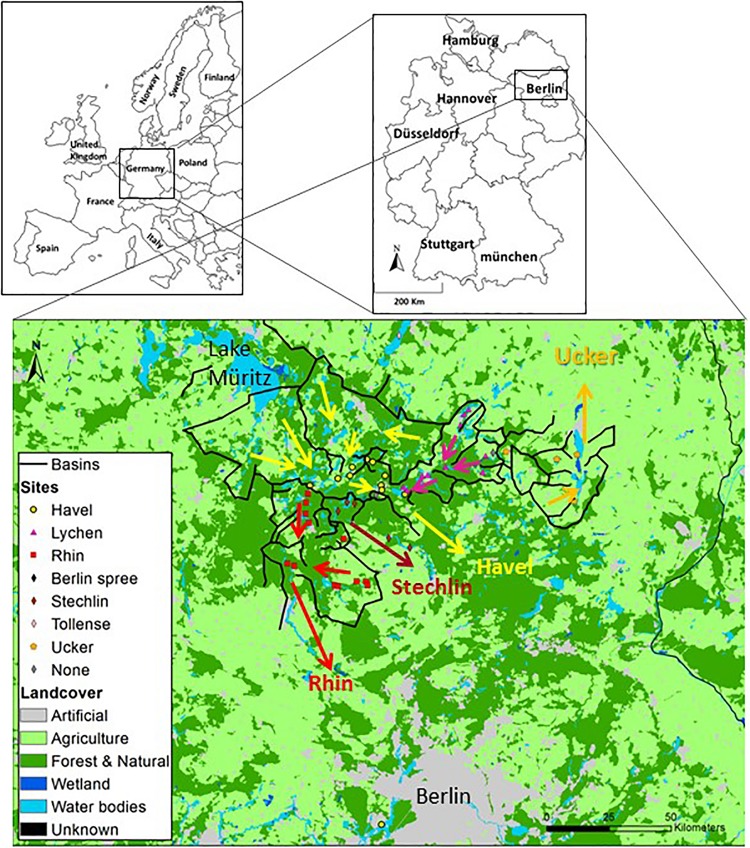
Land use and drainage basins in the sampling area. A total of 46 samples were collected from lakes and a fish-pond in the Brandenburg region of northern Germany. Five types of land use: urban areas, agriculture (further divided for GLM analysis to heterogeneous agriculture, pasture lands and arable lands), forests, wetlands and water bodies, were mapped using GIS. Superimposed black lines connect topographically high points and indicate the borders of the drainage basins and of major sub-basins. The arrows indicates the dominant flow direction within each basin. A map with the identities of each sampling location is presented in Supplementary Figure 1, and the detailed information on each sampling location can be found in Supplementary Data File 1.

In this study, we investigated the microbiomes of 46 German lakes, located in a network of 7 drainage basins connected through streams. The region encompassing these lakes and basins comprises a tapestry of different natural and artificial land parcels (e.g., forests, pastures, agricultural lands, water bodies, towns, and villages, Figure 1). Our major goal was to understand what shapes microbial population in such a system of connected natural water bodies, located within relatively short distances and surrounded by a variety of different land-use types. We hypothesize that: (1) land use directly affects water chemistry; (2) water chemistry affects bacterial composition by habitat filtering of bacteria adapted to specific water properties; (3) the similarity of bacterial populations structure will gradually decay with distances, as the water moves downward within the connected lakes and streams; (4) land use directly affects the microbial populations in the water via direct input of live bacteria.

## Materials and Methods

### Study Region and Delineation of Drainage Basins

The sampling area lies within the region of Brandenburg, in northern Germany (Figure 1, Supplementary Figure 1, and Supplementary Text). The area is relatively flat (18–89 m above sea level) and contains hundreds of lakes, ponds and depressions that were formed by glacial action during the Pleistocene. Four main basins drain this region, which are (from west to east) the Rhin, the Stechlin and the Havel, all of which flow south, and the Ucker, which flows north. These four main basins can be subdivided into smaller basins. The drainage basin of each lake was defined as the line connecting the highest points from which the surface water flow enters each of the lakes, including all of the upstream lakes. We note that groundwater flow may be significant in many lakes, and often does not flow as surface water does in accordance with topographic basins (i.e., the direction of flow may be different, Winter et al., 2003). We also assume that the water is fully mixed horizontally within each separate water body, and that our sampling represents the epilimnion if the lakes are stratified. A detailed description of the sampling region, methods of delineating the basins, identification of the canals connecting the basins and GIS layer analysis, can be found in the Supplementary Information.

### Sampling and Analysis of Environmental Data

Each location was sampled once from the edge of the water body, as due to the range of water bodies sampled and the inaccessibility to some by boat, sampling from the deepest point was not feasible. An exception are several lakes that were sampled from a boat as part of the IGB routine monitoring program (Stechlin, Schmaler Luzin, Feldberger Haussee, Breiter Luzin, and Tollensesee). When possible, we sampled from a pier or other location where relatively deep water was accessible. When sampling had to be performed from the littoral, water was sampled from a depth of at least 0.5 m, where no water movement was observed, and care was taken not to resuspend any sediment material. During sampling, dissolved oxygen, temperature pH and turbidity were measured using a YSI multi probe (YSI 6920 V2-2; YSI Inc). At all locations, 5 l of surface water were collected using plastic jerry-cans that were washed with double distilled water then with 70% ethanol and air dried prior to sampling. Care was taken not to raise any sediment. Samples were brought to the lab and filtered within 2 h. For *chlorophyll a (chl* a) measurement, water was filtrated on GF/C filters (Whatman glass fiber, 25 mm, nominal pore size 1.2 μm). The filters were placed in 1.5 mL sterile Eppendorf tubes. The water was filtered until the filters were clogged, and the volume was recorded (50–800 mL). *Chlorophyll a* was extracted in absolute methanol for 12 h at room temperature in the dark, and the concentration was determined spectrophotometrically according to (Ritchie, 2008). For DNA extraction, water was filtered using a vacuum pump on GF/F filters (Whatman glass fiber, 25 mm, nominal pore size 0.7 μm). The reason for choosing this filter type is that the study presented here is part of a larger study in which microbial populations were assessed using both metabolomic and genetic methods. Metabolomic analyses require extraction with organic solvents, to which the 0.22 mm filters often used for 16S analysis are not resistant. Preliminary experiments using a culture of the marine Pico-Cyanobacterium *Prochlorococcus* MED4 (∼0.5 μm in diameter) showed that with the vacuum pumps we used approximately 99.9% of the cells of this size were retained on a GF/F membrane (see discussion for specific caveats of this approach). The filters were placed into 1.5 mL sterile Eppendorf tubes with 1 mL of storage buffer (40 mM EDTA, 50 mM Tris-HCl, 0.75 M Sucrose) and stored at −80°C until analyzed. The filtrate from the GF/F filters was collected for measurement of dissolved nutrients, and unfiltered water was used for total phosphorus measurements according to (Grasshoff and Kremling, 1983; Grasshoff et al., 2009). DNA extraction was performed using bead-beating followed by robotic extraction using a QiaCube robot, the gene encoding the 16S subunit of the ribosomal RNA was amplified using primers targeting the V3-V4 region, and the amplicons were sequenced using the 2 × 250 base pair format on a MiSeq flow cell (V3 chemistry). More details on the DNA extraction (including some important caveats regarding the possibility of cross contamination) and 16S amplification procedures are found in the Supplementary Methods Section. The OTU table can be found in Supplementary Data File 1.

### Generalized Linear Model Analysis (GLM)

We used multivariable regression model in the framework of GLMs to relate the six land use types to the measured water chemical parameters. Multi-model inference based on the Akaike Information Criterion (AIC) was used to rank the importance of variables (Burnham and Anderson, 2003; Blank and Blaustein, 2014). The package “glmulti” was used to facilitate multi-model inference based on every possible first-order combination of the predictors in each scale (Calcagno and de Mazancourt, 2010). The coefficients associated with each variable and their relative importance were assessed using a multi-model average. Parameters were estimated by averaging across models, weighted by the probability that their originating models are the best in the set. Hence, the relative importance of a parameter is not based on *P*-values, but rather on its occurrence in the models that are best supported by the data. The importance value for a particular predictor is equal to the sum of the weights for the models in which the variable appeared. Thus, a variable that shows up in many of the models with large weights received a high importance value.

### 16S Sequence Analysis and Statistical Tests

Sequences received from the sequencing facility were quality filtered to remove PhiX DNA, unmerged reads and low quality sequences, followed by classification into different Operational Taxonomic Units (OTUs) based on 97% sequence similarity threshold using the “pick_de_novo_otus.py” command in Qiime (Caporaso et al., 2010). OTUs with fewer than 5 reads across all samples were removed, resulting in 35,775 – 75,704 reads per sample. For all the statistical analyses, the OTU table was subsampled to 25,000 reads for each location and were then log10 transformed. Results were very similar when compared to un-transformed OTU table. Alpha diversity (Shannon index) was calculated using the “diversity” function, and Bray–Curtis dissimilarity by “vegdist” function. Non-metric multidimensional scaling analysis (nMDS) was carried out in R with “metaMDS” function and a Bray-Curtis matrix. Results were very similar when a non-quantitative distance metric (Jaccard) was used. In order to relate environmental factors to specific OTUs we performed variation partitioning (“varpart” function in R) followed by canonical correspondence analaysis (CCA) using the “cca” and “anova.cca” functions in the “vegan” R package. For additional information on these analyses, see the Supplementary Methods. The sequences are available in the GenBank as BioProject PRJNA483954 (Sequence Reach Archive accession SRP155981).

### Permutation Test of Phyla Clustering Significance

In CCA ordinations, the location of each OTU within the ordination is related to the effect of the variables constraining the CCA on that OTU. We wanted to address a different question – whether a group of OTUs (e.g., a phylogenetic lineage) behave coherently with respect to the environmental characteristics. In other words, we do not ask, for example, whether “a cyanobacterial OTU” is related to the CCA axes (as that is the direct output of the CCA), but whether the cyanobacteria as a group behave similarly with respect to these axes. We therefore designed a permutation test to determine: (a) the clustering significance of the OTUs within each clade; (b) the significance of association with the tested environmental factors. We obtained the coordinates of eigenvectors (e.g., pH, temperature, etc.), and of all OTUs belonging to the different clades, in the CCA data. For each clade *p*, we calculated two scores: *d*_*p*_ and *d*_*p,v*_, where *v* is a given eigenvector. The score *d*_*p*_ equals the sum of Euclidean distances between all pairs of OTUs in *p*. The score *d*_*p,v*_ is the sum of scalar projections of all OTUs in *p*, on a given eigenvector v. Accordingly: (a) for the clustering test, *s*_*p*_ OTUs were randomly chosen from all tested clades, to obtain the score *d*_*p=random*_, where *s*_*p*_ is the count of OTUs in clade *p*. This calculation was repeated n times (*n* = 1000), where the count of cases where *d*_*p*_ ≥ *d_p = random_*, divided by *n*, was defined as *p*-value; (b) for the association test, The score *d*_*p,v*_ was compared to the score *d*_*p* = *random,v*_ which was obtained from random coordinates normally distributed with the same x,y variance as the x,y variance as in clade *p*, and the same number of *s*_*p*_ datapoints. This calculation was repeated n times (*n* = 100), where the count of cases with *d*_*p.v*_ ≥ *d*_*p* = *random,v*_ divided by *n*, was defined as *p-*value. Here, *p*-value < 0.025 or >0.975 (after Benjamini–Hochberg correction for multipole hypothesis testing) indicates negative and positive association with the tested environmental factor.

## Results

### General Characterization of the Sampled Water Bodies

**FIGURE 2:**
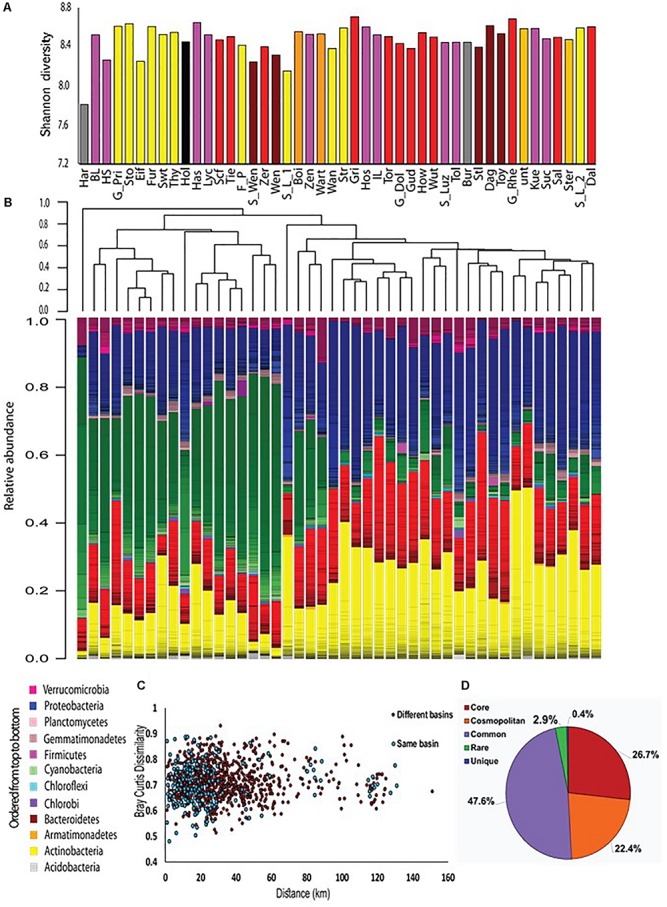
An overview of the microbial populations in the sampled lakes. **(A)** α-diversity of the microbial populations in the different lakes, measured by the Shannon index. The columns are colored by drainage basins. No clear patterns are observed. **(B)** Community composition and community clustering based on the Bray–Curtis similarity index; Colors represent different phyla, lines within the same color represent OTUs at the 97% identity cutoff. The sampling sites can be divided into two groups: one dominated by Cyanobacteria, and the other by Actinobacteria, Proteobacteria and Bacteroidetes. **(C)** No distance decay patterns are observed. Each point represents the Bray–Curtis dissimilarity between locations within the same basin (blue) or locations found in two separate basins (red). Spearman’s *r* between geographic distance and Bray–Curtis distance is 0.03, *p* = 0.30. **(D)** Most of the OTUs are found in more than 50% of the sampling sites. The diversity (γ diversity) of all sampling locations combined is presented, divided into five categories representing core OTUs (found in all sites), cosmopolitan OTUs (>90% of the sites), common OTUs (>50% of the sites), rare OTUs (<10% of the sites), and unique OTUs (present at one site only).

During the summer of 2015, we sampled 46 water bodies (lakes and one fish pond) within a relatively small area (∼150 km by 100 km) of the Brandenburg/Mecklenburg lake district in northeastern Germany (Supplementary Data File 1). The lakes ranged in trophic status from meso-oligotrophic (Lake Stechlin) to polytrophic (hyper-eutrophic), with total phosphorus concentrations between 0.009–0.589 mg/L and *chl* a concentrations ranging between 0.0005 and 0.320 mg/L. The water bodies belonged primarily to five major drainage basins (Figure 1 and Supplementary Figure 1), and varied widely in their sizes and nutrient concentrations (Supplementary Data File 1). While the microbial diversity, expressed as the Shannon alpha-diversity index, between the basins was relatively similar (4.88–7, Figure 2A), clear differences were observed in community composition between the various lakes (Figure 2B). Comparing the microbiome composition between lakes (using the Bray–Curtis dissimilarity index) revealed that they can be broadly divided into two main groups: a cluster of sites with a high relative abundance of Cyanobacteria, and a second cluster with a high relative abundance of Actinobacteria, Proteobacteria and Bacteoidetes and low relative abundance of Cyanobacteria (Figure 2B).

Lakes are often broadly divided into trophic states, using parameters such as *chl* a or total phosphorus (TP) concentrations, and these trophic definitions are often used in water management. As shown in Supplementary Figure 2, the trophic status of the lakes based on *chl* a and total P concentrations was broadly in agreement with estimates based on multi-annual monitoring by the German federal states of Mecklenburg-Vorpommern and Brandenburg, although more of the lakes had an oligotrophic character at the time of sampling. This is likely because we sampled during early summer where high production rates of zooplankton can reduce phytoplankton abundance, leading to a “clear water phase” and masking the real, higher trophic status of lakes high in nutrients. A tendency was seen for lakes with higher *chl* a and total P concentrations to have a higher relative abundance of Cyanobacteria (Supplementary Figure 2) manifested also as an observable trend in the NMDS ordination (Supplementary Figure 3A).

Interestingly, lakes from the same drainage basins did not cluster together, despite relatively large differences between the drainage basins in terms of their size and land-use in their watershed (Figure 2B, Supplementary Figure 3B, and Supplementary Data File 1). Neither was a clear correlation observed between size of the drainage basin of each water body and chemistry of the water (Supplementary Figures 4A–F) or lake microbiome (Supplementary Figure 4G). Finally, no relationship was seen between the location of sampling (littoral or by boat) and the microbiome structure (Supplementary Figure 3C).

One major factor, that might determine to what extent ecological communities differ, is the geographical distance between them. This phenomenon, identified by a “distance decay” relationship in community similarity (Nekola and White, 1999; Bell, 2010), has been demonstrated for both macro- and micro-organisms at various scales of geographic distances (Lear et al., 2013; Marcelino et al., 2017). No distance decay relationships were observed in our samples, neither within a single basin nor between samples belonging to different basins (Figure 2C). The lack of distance-decay patterns was still observed when the three major basins, Lychen, Rhin and Havel, were divided into sub-basins where all of the water bodies were directly connected (Supplementary Figure 5). Moreover, examination of the total species diversity in our landscape (i.e., γ-diversity) showed that 27% of the OTUs are core OTUs that were present among all sites, and that additional 22% OTUs were cosmopolitan, defined as being observed in more than 90% of all sites (Figure 2D). Rare (i.e., present in less than 10% of the sites) and site unique OTUs (i.e., present only in one specific site) represented together only 3.4% of all OTUs. These rare and unique OTUs were also numerically scarce, representing at most 2.6% of the total sequence reads. The core microbiome was dominated by Actinobacteria and Proteobacteria (43 and 39% of the OTUs respectively, Supplementary Figure 6A), and contained very few Cyanobacteria OTUs (1%). In contrast, Cyanobacteria represented the dominant phylum of all cosmopolitan OTUs (47%). Thus, it seems that differences between microbial communities are primarily due to changes in the relative contribution of OTUs rather than to the presence of unique or endemic OTUs in different regions.

### Land Use in the Drainage Basins Affects Water Chemistry, and Through it, the Microbiome

**FIGURE 3:**
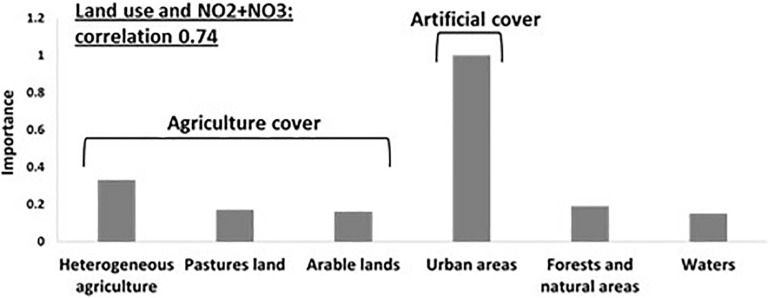
Specific land use type affects aquatic environmental parameters: General Linear Models (GLMs) analysis were performed and resulted in Urban areas are positively related to NO_2_ + NO_3_ concentrations. A strong correlation (*R*^2^ = 0.74) is observed between land uses and nitrite and nitrate concentrations, with the fraction of “urban areas” being the most important variable contributing to the correlation.

We next asked whether specific land use types may affect environmental conditions within the water body (e.g., nutrients, temperature, etc., referred to here as “aquatic environmental conditions,” see Supplementary Information for the definition of the land-use types used). The effect of land use could be manifested at the level of the entire drainage basin (summing up the input of the entire draining into each water body), or, alternatively, at the level of local land use (adjacent to the water body). To test our hypotheses, we used General Linear Models (GLMs) to determine whether specific land use types could be associated with specific aquatic environmental conditions. On the basin scale, six land use types (agricultural area, pasture lands, arable lands, urban and forest areas and water bodies) explained a high proportion of the variability of nitrite and nitrate concentrations (NO_2_ + NO_3_, *R*_*N*_^2^ = 0.74, Figure 3 and Supplementary Table 1), with the fraction of urban land being positively correlated to NO_2_ + NO_3_ concentrations (Supplementary Table 1). When quantifying the independent contribution of each different land use separately, urban was the only significant variable explaining 74% of the variation in NO_2_ + NO_3_.

**FIGURE 4:**
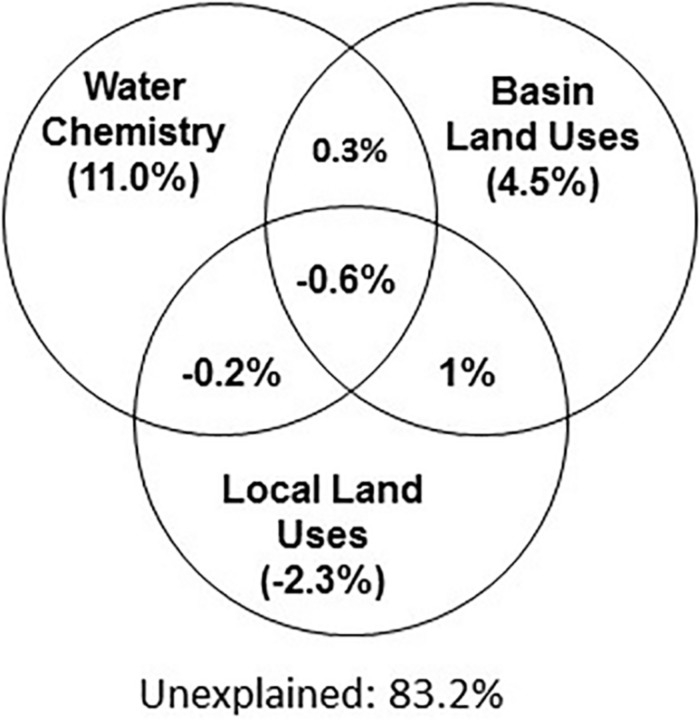
Water chemical properties represent the main explanatory variable for the microbiome composition (ca. 11% among all sites). Variation Partition Analysis performed with 3 data sets (basin and local land use as well as water chemistry) to explain the variation in the microbiome composition. The combination of land uses and water chemistry explain 16.8% of microbiome variability. See Supplementary Information for an explanation of the estimation of explained variability.

**FIGURE 5:**
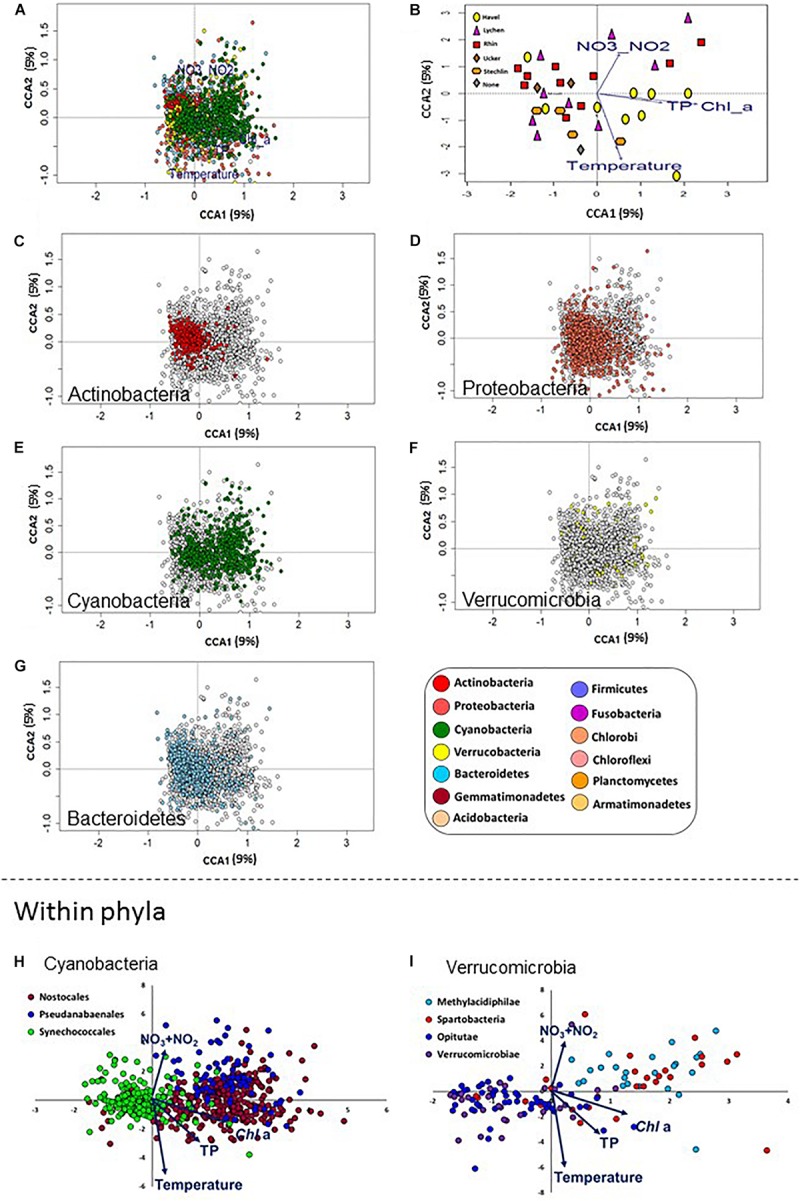
Differences between bacterial phyla in their association with different aquatic environmental conditions. Canonical Correspondence Analysis (CCA) plots are shown, with sampling locations and OTUs ordinated based on the four aquatic environmental parameters shown to significantly affect the microbiome composition. **(A)** An overview of the ordination, showing the 2845 most abundant OTUs. The following panels each highlight a different aspect of this ordination. **(B)** The same ordination as in A, with the OTUs removed to show the relative location of the eigenvectors for the aquatic environmental parameters and the ordination of the sampling sites. **(C–G)** The same plot as in **(A)**, with specific microbial phyla highlighted. **(H,I)** Separation in the CCA ordination between selected lineages within the Cyanobacteria (h, orders) and Verrucomicrobia (I, classes). Note the clear separation between the Synechococcales and the Pseudanabaenales and Nostocales along the first CCA axis with some differentiation between the latter two orders primarily along the second CCA axis (h). Within the Verrucomicrobia, the Methylacidiphilae, which are limited to the upper-right hand quadrant, possibly in association with high NO_2_ + NO_3_ levels, are clearly separated from the other orders, except of the Spartobacteria.

We next used Variation Partition Analysis (VPA) to explore whether and to what extent basin-level and local-level land use types, as well as aquatic environmental conditions, explain the composition of the aquatic microbiome. VPA results pointed to aquatic environmental parameters as key parameters, in total explaining 16.8% of the variance in microbiome composition among all sites, 11% independent of land-use parameters (Figure 4). In contrast, land-use types (at both basin and local scale) had a very weak explanatory power for microbial community structure (Figure 4). Following VPA results, we tested measured aquatic environmental parameters as explanatory model for community composition using CCA followed by CCA permutation test (Supplementary Table 4 and Figure 5). The aquatic environmental parameter model explained 24.2% of the inertia in CCA and was significant (*P* < 0.05). Similar to the NMDS (unconstrained) ordination (Supplementary Figure 3A), the CCA did not show clear separation of sampling sites by basin (Figure 5B). Four out of six aquatic environmental parameters in our model were significant: total phosphorus (TP, *P* < 0.01) and *Chl a* (*P* < 0.05) concentration were positively associated with CCA1 (eigenvector for CCA1 and CCA2 were 0.89, −0.12 for *Chl a* and 0.45, −0.16 for TP), NO_2_ + NO_3_ (*P* < 0.05) was positively associated with CCA2 (eigenvector 0.30, 0.58) and water temperature (*P* < 0.01) was negatively associated with CCA2 (eigenvector 0.08, −0.84) (Figure 5A and Supplementary Table 4). Notably, NO_2_ + NO_3_ were identified by the GLMs as being significantly affected by land use in the drainage basins.

### Specific Bacterial Taxonomic Groups Are Associated With Sampling Locations Differing in Their Aquatic Environmental Conditions

Having identified TP, *Chl* a, NO_2_ + NO_3_ and temperature as variables affecting the microbiome as a whole, we next asked whether we could identify specific microbial lineages affected by these environmental conditions. To answer this question, we analyzed the distribution patterns of OTUs belonging to different bacterial lineages along the CCA ordination axes. Most phyla were widely distributed in the CCA biplot (Figure 5), suggesting that, within each phylum, OTUs were affected differently by environmental factors driving the CCA ordination. However, Actinobacteria OTUs formed a tight cluster whose center was on the negative side of the *x*-axis (Figure 5C). This suggested that all OTUs in this group were affected similarly by the measured environmental variables. To statistically test this, we developed a permutation test to determine: (a) whether OTUs in each lineage were clustered together more than expected (i.e., the distances between them were smaller than those between randomly selected OTUs. This suggests a coherent relationship with the factors constraining the CCA at the lineage level); (b) whether the OTUs were associated (positively or negatively) with any of the eigenvectors of the specific aquatic parameters (i.e., the arrows in Figure 5B). We then applied these two tests to lineages at different phylogenetic resolution (phylum, class, order, and family) to identify those that are coherently affected by the four environmental parameters tested (Supplementary Data File 2).

Of the five most abundant phyla, only Actinobacteria were not randomly distributed in the CCA plot, as determined by permutation analysis (Figures 5C–G, *P* < 0.05, Supplementary Data File 2). As a phylum, they were negatively associated with *Chl* a, temperature and total P. This suggests that, while the relative abundance of Actinobacteria was different between different sites, within this phylum OTUs behaved similarly, i.e., they are found at similar ratios at each location (Figure 2B). Proteobacteria showed a wider distribution range that was close to significance in our test (adjusted *p*-value = 0.066, Figure 5D). These results are in agreement with our observation that Actinobacteria and Proteobacteria OTUs dominate the core microbiome of all sites (Supplementary Figure 6A) and that within each of these phyla, a relatively large fraction of OTUs was core or cosmopolitan (Supplementary Figure 6B). In contrast, Verrucomicrobia, Cyanobacteria and Bacteroidetes did not cluster together in the CCA plot (although, as phyla, they were associated with some water parameters (Figures 5E–G and Supplementary Data File 2).

At a finer phylogenetic resolution, several lineages clustered in a non-random way and correlated with the tested parameters (Supplementary Data File 2). In some cases, different lineages within the same phylum behaved similarly. For example, the families C11 (order Acidimicrobiales) and ACK-M1 (order Actinomycetales), both belonging to the Actinobacteria, behaved similarly, being negatively associated with *Chl* a and total P concentrations, as well as with temperature. However, in other cases different lineages clearly clustered separately and were affected differently by the tested environmental factors, suggesting potential niche differentiation. Within the Cyanobacteria, the order Synechococcales was clearly separated in the CCA ordination from the Pseudoanabaenales and Nostocales (Figure 5H). The former was clustered non-randomly and was negatively associated with *Chl* a, NO_2_ + NO_3_ and TP (Supplementary Data File 2), whereas the two latter orders were positively associated with these factors. Nostocales and Pseudanabaenales, in turn, differed in their correlation with temperature (positive and negative association with temperature, respectively). Notably, while Cyanobacteria appeared at all sampling sites (Figure 2B), few Cyanobacteria OTUs were part of the core microbiome (Supplementary Figure 6A), yet this phylum had the highest fraction of OTUs in the “common” category among all analyzed phyla (Supplementary Figure 6B). This further suggests that different Cyanobacteria of different water bodies, potentially correlated with water quality and trophic status. Differences in the ordination of groups at the class level were also observed within the phylum Verrucomicrobia, with Opitutae clustered non-randomly and associated with low NO_2_ + NO_3_ and *Chl* a, clearly separating from other classes such as Methylacidiphilae and Spartobacteria, both of which were positively associated with *Chl* a (although not clustered, Figure 5H). Additional correlations of potential interest are found in Supplementary Data File 2.

## Discussion

**FIGURE 6:**
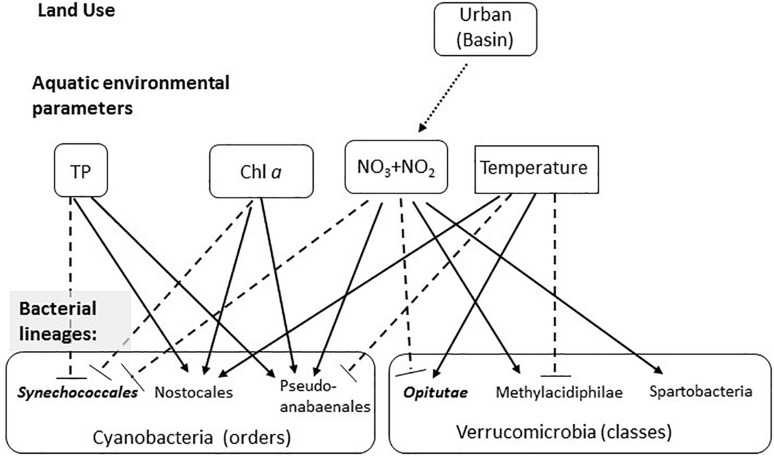
Conceptual model summarizing the observed effects of land use on water chemistry, and of water chemistry on selected microbial lineages. The first layer represents the effect of land use on aquatic environmental parameters (based on the GLMs), with the arrow showing the significant correlation between urban land and NO_2_ + NO_3_. The seconds layer shows the effect of the four environmental parameters shown to significantly affect the microbiome structure (based on the CCA analysis) on selected microbial linages within the Cyanobacteria and Verrucomicrobia. Solid arrows show significant positive correlations (based on the permutation analysis), dotted blunt-ended lines show negative correlations. Lineages that are more clustered than randomly expected are in bold and italics.

The quality and chemical properties of freshwater ecosystems, aquatic microbial populations, and human health are tightly interlinked. In this study, we analyzed the microbiomes of a network of partly interconnected lakes, which belong to several different drainage basins. We aimed to identify to what extent land-use in the drainage basins of the lakes affects both environmental conditions in the water and lake microbiomes, posing four hypotheses: (1) land use directly affects water chemistry; (2) water chemistry affects bacterial composition by habitat filtering of bacteria adapted to specific water properties; (3) the similarity of bacterial populations structure will gradually decay with distances, as the water moves downward within the connected lakes and streams; (4) there may be also a direct effect of land use types on the water microbiome via direct input of live bacteria. Several previous studies have identified correlations of land-use to various parameters of water chemistry, as well as to the abundance of specific members of the microbial community (Savage et al., 2010; Katsiapi et al., 2012; Beaver et al., 2014; Davis et al., 2015). However, few studies have attempted to determine whether land use controls the microbiome structure as a whole (Glöckner et al., 2000; Van Rossum et al., 2015). Importantly, in our analyses, we used tools that are relatively accessible to water quality managers: land-use maps are available for many regions of the world, and are often used for land management using GIS (Joerin et al., 2001; Zhang et al., 2015). Many of our measurements were performed using hand-held instruments, and measurements of inorganic nitrogen compounds and total phosphorus were performed using commonly applicable and standardized methods (Grasshoff and Kremling, 1983; Grasshoff and Ehrhardt, 1999; Louca et al., 2016). Our results suggest a conceptual model (schematically presented in Figure 6) whereby NO_2_ + NO_3_ is related to a specific land use within the drainage basin of each water body (urban areas). In turn, NO_2_ + NO_3_, together with total phosphorus, *chl* a and temperature, are related to the relative abundance of specific microbial linages at different phylogenetic resolutions.

Below we first discuss specific links between land use types, water quality, and microbiome composition, as well as the association between specific lineages and water quality parameters. We then mention the caveats of our study and suggest ways to improve future studies.

### Indirect Effect of Land Use on the Aquatic Microbiome, Through Water Chemical Properties

Several studies have investigated the association between land use types and water properties (Quinn et al., 1997; Larned et al., 2004; Mehaffey et al., 2005; Schoonover and Lockaby, 2006; Reimann et al., 2009). These studies generally suggest higher dissolved nitrogen and phosphorus concentrations in waters surrounded by high coverage of urban areas and agriculture lands (Wang and Yin, 1997; Ghai et al., 2014). In our study, urban land was an important predictor for (and positively correlated with) concentrations of NO_2_ + NO_3_ (Figures 3B,C and Supplementary Tables 1, 2). The correlation between urban area (which includes also industry) and NO_2_ + NO_3_ concentrations is in agreement with the previously mentioned studies, and may be driven by discharge of waste and rain run-off from sealed surfaces, both containing high nitrogen levels. Unlike previous studies, which observed a negative correlation between forested area and total phytoplankton as well as Cyanobacteria biomass (Catherine et al., 2008), we did not observe such an effect, despite having sampled in lakes encompassing a wide array of trophic states. Neither did we observe an expected effect of agricultural land, e.g., through the effect of nutrient runoff (Carpenter et al., 1998; Xu et al., 2010), potentially because this region is characterized by low P fertilizer application and low livestock densities (Theobald et al., 2016). Thus, in the region we studied, no clear correlation was observed between land use and total P or *chl* a concentrations (the low number of oligotrophic-mesotrophic lakes precluded an analysis of the trophic levels themselves). We note, however, that the interaction between land-use in lake catchments and the physics, chemistry and biology of the lakes is complex, with potentially conflicting drivers. For example, nutrients and dissolved organic matter exported from, e.g., forests into lakes may, on the one hand, promote phytoplankton and bacterioplankton growth, while at the same time increasing water color and subsequent light attenuation could result in changes in lake stratification (reviewed by Solomon et al., 2015).

In our study, NO_2_ + NO_3_, total phosphorus, *chl* a and temperature were correlated with changes in microbial community structure. Comparing these observations with other studies of freshwater environments, it is clear that the specific environmental parameters affecting microbial community structure may vary between studies, freshwater systems and the time of sampling (e.g., Crump et al., 2007). Temperature is often identified as an explanatory variable (Van der Gucht et al., 2007), but this is not always the case (Savage et al., 2010). Similarly, *chl* a and total P concentrations are also sometimes identified as important variables (Yannarell and Triplett, 2005), but not always (Crump et al., 2007; Van der Gucht et al., 2007; Niño-García et al., 2016), and these two parameters may have a stronger effect on particle-associated rather than free-living bacteria (Savio et al., 2015). In our dataset, *chl* a and total P concentrations (which are often used as quantitative measures of lake trophic state) were somewhat related to the relative abundance of Cyanobacteria (Supplementary Figure 2), especially Nostocales and Pseudanabaenales (Figure 5). While pH is often identified as important (Yannarell and Triplett, 2005; Crump et al., 2007; Savio et al., 2015; Niño-García et al., 2016), it was not a statistically significant explanatory variable in our analysis. Finally, consistent with our results, NO_2_ + NO_3_ have been shown to affect community structure in some cases (Kozak et al., 2019), whereas in other studies total N (which was not measured here) was shown to be important (e.g., Van der Gucht et al., 2007).

The potential effect of water properties on the microbiome structure was quantitative rather than qualitative, resulting in differences in the relative abundances of different microbial groups or OTUs rather than driving some of them locally extinct (Supplementary Figure 6). Indeed ∼50% of the OTUs were found in >90% of the sampling locations, suggesting a “core” microbiome that is found at all sites, with different relative abundances (Savio et al., 2015). In organism-associated microbiomes, it has been suggested that microorganisms that are commonly found across locations or individuals (thus forming the core microbiome) may be critical to the function of the microbial community, and indeed may define a “healthy” community (Shade and Handelsman, 2011; Hernandez-Agreda et al., 2017). Such studies are still rare for aquatic microbiomes, yet it is possible that better understanding of the dynamics of the core microbiome may help understand how microbial communities are related to – and affect – water quality.

While we were able to explain, to some extent, water parameters via land-use types, and the microbiome composition via water chemistry, we were unable to identify direct links between land use and the specific microbiome composition. In a study of water bodies in the Ile-de-France region (Catherine et al., 2008), land use was able to partly explain Cyanobacteria biomass, and in a subsequent study land use could predict to some extent the phytoplankton structure through the effect of land-use on water quality (Catherine et al., 2016). Similarly, a land-use based model was able to partly predict the presence of genes involved in the biosynthesis of the Cyanobacteria toxin microcystin (Marmen et al., 2016). In our dataset, even though the total fraction of Cyanobacteria is correlated with *Chl a* (*R*^2^ = 0.3, Supplementary Figure 3C), and the main groups of Cyanobacteria partition clearly into different niches based on the water chemistry (Figure 5H), we were unable to identify any land-use based model that could explain the variation in the relative abundance of these specific Cyanobacteria groups. Thus, while the potential of land-use to predict the abundance of Cyanobacteria and their toxins has been demonstrated, further work is needed to develop the appropriate methodologies and statistical tools that will identify linkages between land use types and microbiome structure. Finally, it is possible that land use might affect the function of the microbial population (at the genetic level) but that such an effect would not be clearly seen in the population structure, as measured by 16S amplicon sequencing (e.g., Louca et al., 2016).

### Biogeography and Bacterial Mass Transfer Do Not Determine the Microbiome Composition of the Sampled Lakes

Our initial hypothesis was that two types of factors would structure the microbial communities: (i) environmental conditions within the water body, which are partly determined by land use within the drainage basin of each water body (often termed “species sorting,” e.g., Van der Gucht et al., 2007; Logue and Lindström, 2010), and (ii) mass effects due to bacterial transport along the streams connecting the various sampled lakes. The relative importance of these two processes differs between different freshwater systems, with mass effects generally thought to be more prevalent in streams or smaller lakes with low water retention time and species sorting more prevalent in larger lakes with higher water retention times (Mašín et al., 2003; Lindström and Bergström, 2004; Crump et al., 2007; Logue and Lindström, 2010; Niño-García et al., 2016). However, not all lake systems are alike, for example, distance-decay relationships (which suggest mass effects) were observed in some lake systems (e.g., Van der Gucht et al., 2007; Nelson et al., 2009), but not in others (e.g., Niño-García et al., 2016). Contradicting our hypothesis, no separation of bacterial communities by basin and no distance-decay patterns was observed among the German lakes studied (Figure 2C and Supplementary Figure S2A). We note, however, that we did not measure the actual flux of water between the lakes, which could strongly affect the rate of emigration between the lakes (see “caveats,” below). Given the currently available data, it seems likely that, at the geographic scale and time-points we studied, there is no clear limitation on the dispersal of bacteria between the lakes. Rather, environmental conditions within each water body affected to some extent the microbiome structure, and these were partly determined by land-use in the drainage area of each water body (Figure 4).

Another way in which mass transfer from terrestrial habitats could affect aquatic microbial communities is through the inoculation of lakes with live bacteria from the surrounding soil. If this was the case, we would expect such soil bacteria to be present in the water at concentrations that will be detected by sequencing. However, relatively few OTUs belonged to Acidobacteria (Supplementary Figure 6A) regarded as a representative phylum of soil bacteria (Janssen, 2006). Among these, only 0.04% of the total reads belonged to the *Solibacteres*, which have been considered as a marker for soil bacteria (Battistuzzi and Hedges, 2009). Thus, this direct land-to-water microbial link is likely weak in the German lakes during the time of sampling.

### Niche Differentiation of Freshwater Bacterioplankton at the Class and Order Levels

An important question in microbial ecology is whether there is a phylogenetic resolution at which environmental conditions can be best related to microbiome structure and function. Many studies focus on the phylum level (Lindström et al., 2005; Briée et al., 2007; Wolz et al., 2018), since, at a broad scale, bacterial phyla differ in some basic functional traits including their ability to photosynthesize, or to utilize different carbon sources (Mitsui et al., 1986; Op den Camp et al., 2009; Newton et al., 2011). Indeed, a clear differentiation had been observed, in our dataset, between two groups of sites, those with a high relative abundance of Cyanobacteria, and those with a high relative abundance of Actinobacteria and Proteobacteria (Figure 2B). However, it has been suggested that this resolution is far too coarse to reliably infer community function, due to the high genomic and functional variation within phyla and even within individual bacterial lineages (Newton et al., 2011; Singer et al., 2016). Our results suggest that some bacterial lineages clearly partition between different environments at low phylogenetic resolution, namely that of bacterial classes and orders. Several of these patterns were in agreement with our previous expectations based on literature data. For example, in freshwater environments, it is generally accepted that the relative contribution of Pico-Cyanobacteria (e.g., Synechococcales) to total phytoplankton decreases with higher trophic status. However, temporal patterns can be also important (Fukushima et al., 2017). This may be attributed to the ability of Pico-Cyanobacteria to acquire major nutrients and trace metals at sub-micromolar concentrations, a capability that enables them to occupy relatively oligotrophic niches (Callieri, 2017). Larger phytoplankton (e.g., Nostocales) fills in niches with higher nutrient concentrations, and hence are physiologically adapted to proliferate at rich nutrient waters like fishponds and hypertrophic reservoirs (Cires and Ballot, 2016). The presence and density of Nostocales are of great interest from a water quality perspective due to their ability to produce a variety of secondary toxic metabolites (Sukenik et al., 2012). In our samples, a clear separation could be observed in the CCA analyses between Synechococcales, associated with conditions of low *Chl a* and TP, and Nostocales, associated with the opposite conditions (Figure 5H). Pseudanabaenales, which are filamentous Cyanobacteria whose cells are smaller than those of the Nostocales, were associated also with high NO_3_ + NO_3_ concentrations. The physiology and ecology of Pseudanabaenales are relatively poorly known (Acinas et al., 2008), yet these organisms are quite dominant in many of the lakes we sampled (Supplementary Figure 6), providing an incentive for further studies. The niche differentiation between Synechoccocales, Nostocales and Pseudanabaenales is the process that underlies the relative scarcity of ubiquitous Cyanobacteria OTUs, which instead dominate the “cosmopolitan” group (Supplementary Figure 6), and further highlight the potential importance of understanding the dynamics of “core” microbes in relation to water quality.

Potential niche differentiation was also observed within the phylum Verrocumicrobia, which is ubiquitous but relatively less studied in freshwater environments (Figure 5I, Newton et al., 2011). Some members of this phylum were found to be associated with high nutrient concentrations or algal blooms (Eiler and Bertilsson, 2004; Kolmonen et al., 2004; Haukka et al., 2006). In our study Methylacidiphilae (and possibly also Spartobacteria) clearly separated from the other three classes (Pedosphaerae, Opitutae and Verrucomicrobias), and may be associated with high NO_2_ + NO_3_ concentrations and low temperatures. At least some members of the Methylacidiphilae are methanotrophs (Op den Camp et al., 2009), and one bacterium from this class (*Methylacidiphilum fumariolicum*) is a diazotroph, able to actively fix atmospheric N_2_ (Khadem et al., 2010). While most methanotrophic Verrucomicrobia have been isolated in extreme hot and acidic environments, the association of potential diazotrophs (Methylacidiphilae as well as Pseudanabaenales) with high NO_2_ + NO_3_ in lakes is intriguing, and additional studies may shed light on their potential contribution to high inorganic N concentrations.

The separation between different orders in the CCA plots suggests that, in freshwater bacteria, niche differentiation may be discernable at intermediate phylogenetic resolution – higher than that of phyla but lower than that of specific species or strains. We also searched for specific OTUs that were associated with aquatic environmental conditions or land use categories, but found very few results, and when correlations were observed, they were relatively weak (maximum Pearson *R*^2^ was 0.53, Supplementary Table 3). Two OTUs, one belonging to Phycisphaerales (phylum Planctomycetes) and one belonging to Saprospirales (phylum Bacteroidetes), were positively associated with NO_2_ + NO_3_ concentrations. Phycisphaerales have been found in ANNAMOX processes (Hu et al., 2015), and Saprospirales are associated with specific soil parameters (King et al., 2010; Hargreaves et al., 2015), yet, the role of these taxa in freshwater is mostly unclear. Thus, in our dataset, no specific OTU can serve as an indicator species for specific aquatic environmental parameters or land uses.

As the amount of genetic data (e.g., 16S rDNA surveys and metagenomes) from freshwater locations rapidly increases, it will be important to explore the best phylogenetic resolution at which to identify correlations between water quality and microbiome structure. Such microbiome data can then be linked to the relative abundance of specific microbial groups such as those involved in nutrient cycling, toxicity or pathogenesis, which in turn are important traits for water quality management.

### Caveats and Limitations

While we could explain a part of the variability in the microbiome (a maximum of 16.8% in the VPA analysis, Figure 4, similar to other studies, Lear et al., 2013), a large part of the variability remains unexplained. Part of this variability may be due to our sampling locations at the edge of the lakes, which may be more strongly affected by weather conditions (e.g., waves leading to sediment resuspension or wind-driven accumulation of floating material at the edge of the water body). Another technical factor which could potentially have affected our analysis is cross-contamination between the sites during the filtration step, which we address only partially through a series of tests and controls described in detail in the Supplementary Information. This variability may also be due to other aquatic environmental parameters we did not measure, for example the quantity and composition of the dissolved organic carbon, which feeds heterotrophic bacteria and clearly has an effect on aquatic microbiomes (Davis et al., 2015; Logue et al., 2015; Wei et al., 2016). Nor did we measure hydrological properties of the systems such as the lake depth, turbulence, stratification or retention times of the lakes, which can also strongly affect microbial population structure (Kopylov et al., 2002; Nogueira et al., 2002; Humayoun et al., 2003; Carini et al., 2005; Lindström et al., 2005; Percent et al., 2008). We also did not include in our analysis the potential contribution of groundwater, even though this might be significant. Lake retention times and potential groundwater inflow, for example, may affect the rate of population change and the relative impact of neutral processes such as emigration compared to effects of environmental drivers. While clearly of importance, often many of these hydrological parameters remain unknown, including for the majority of the lakes we studied, some which have been studied intensively for over 50 years (Allgaier and Grossart, 2006; Casper, 2012). More broadly, we include in our analysis only “bottom–up” controls, whereas biotic interactions such as grazing, phage infection and allelopathy can strongly impact population structure (Louca et al., 2016).

Finally, our sampling approach, based on filtration on GF/F filters, assessed only part of the community, likely under-estimating the relative abundance of ultramicrobacteria (Proctor et al., 2018). Many of these organisms belong to phyla that have yet to be cultured, and understanding their contribution to lake microbial dynamics represents a major future challenge. We also measured each location only once, relatively early during summer. Such a snap-shot does not represent the dynamic nature of the aquatic microbiome. For example, in several lakes that we studied, large Cyanobacteria blooms have been recorded, particularly during August (Mantzouki et al., 2018). These blooms are often dominated by *Microcystis* species, yet these were relatively rare in our samples. Additionally, such blooms might be spatially variable, and thus the coast of the lakes might be affected differently from the center. Clearly, extended time-series are needed to gain a better understanding of the processes affecting aquatic microbiomes in relation to land use. Finally, increasing the number of sampled locations and using land-use maps with higher spatial as well as temporal resolution, may increase the ability to identify specific links between land-use and microbiome structure. Given the good accessibility of land-use data, which for much of the globe (including third world countries) can be derived from remote sensing, exploring further to what extent land use can predict the structure of aquatic microbiomes should be of high priority for future research (Ndlela et al., 2016).

## Data Availability Statement

The datasets generated for this study can be found in the https://www.ncbi.nlm.nih.gov/bioproject/PRJNA483954.

## Author Contributions

SM, H-PG, and DS designed the study. SM, LG, H-PG, and DS collected the samples. LB, LG, H-PG, and DS analyzed the geographic data. SM, LB, AA-A, AM, ML, and DS analyzed the genetic data. SM and DS wrote the manuscript with contributions from all co-authors. All authors have approved the final manuscript for publication.

## Conflict of Interest

The authors declare that the research was conducted in the absence of any commercial or financial relationships that could be construed as a potential conflict of interest.

## References

[B1] Abdelzaher A. M. Wright M. E. Ortega C. Solo-Gabriele H. M. Miller G. Elmir S. (2010). Presence of pathogens and indicator microbes at a non-point source subtropical recreational marine beach. *Appl. Environ. Microbiol.* 76 724–732. 10.1128/AEM.02127-09 19966020 PMC2812993

[B2] Acinas S. G. Haverkamp T. H. A. Huisman J. Stal L. J. (2008). Phenotypic and genetic diversification of *Pseudanabaena* spp. (cyanobacteria). *ISME J.* 3:31. 10.1038/ismej.2008.78 18769459

[B3] Allgaier M. Grossart H.-P. (2006). Diversity and seasonal dynamics of Actinobacteria populations in four lakes in northeastern Germany. *Appl. Environ. Microbiol.* 72 3489–3497. 10.1128/aem.72.5.3489-3497.2006 16672495 PMC1472390

[B4] Battistuzzi F. U. Hedges S. B. (2009). A major clade of prokaryotes with ancient adaptations to life on land. *Mol. Biol. Evol.* 26 335–343. 10.1093/molbev/msn247 18988685

[B5] Beaver J. R. Manis E. E. Loftin K. A. Graham J. L. Pollard A. I. Mitchell R. M. (2014). Land use patterns, ecoregion, and microcystin relationships in U.S. lakes and reservoirs: a preliminary evaluation. *Harmful Algae* 36 57–62. 10.1016/j.hal.2014.03.005

[B6] Bell T. (2010). Experimental tests of the bacterial distance–decay relationship. *ISME J.* 4:1357. 10.1038/ismej.2010.77 20535220

[B7] Blank L. Blaustein L. (2014). A multi-scale analysis of breeding site characteristics of the endangered fire salamander (*Salamandra infraimmaculata*) at its extreme southern range limit. *Hydrobiologia* 726 229–244. 10.1007/s10750-013-1770-8

[B8] Briée C. Moreira D. López-García P. (2007). Archaeal and bacterial community composition of sediment and plankton from a suboxic freshwater pond. *Res. Microbiol.* 158 213–227. 10.1016/j.resmic.2006.12.012 17346937

[B9] Burnham K. P. Anderson D. R. (2003). *Model selection and Multimodel Inference: a Practical Information-Theoretic Approach.* Berlin: Springer Science & Business Media.

[B10] Cabral J. P. S. (2010). Water microbiology. bacterial pathogens and water. *Int. J. Environ. Res. Public Health* 7:3657. 10.3390/ijerph7103657 21139855 PMC2996186

[B11] Calcagno V. de Mazancourt C. (2010). glmulti: an R package for easy automated model selection with (generalized) linear models. *J. Stat. Softw.* 34 1–29.

[B12] Callieri C. (2017). Synechococcus plasticity under environmental changes. *FEMS Microbiol. Lett.* 364:fnx229. 10.1093/femsle/fnx229 29092031

[B13] Caporaso J. G. Kuczynski J. Stombaugh J. Bittinger K. Bushman F. D. Costello E. K. (2010). QIIME allows analysis of high-throughput community sequencing data. *Nat. Methods* 7:335. 10.1038/nmeth.f.303 PMC3156573 20383131

[B14] Carini S. Bano N. LeCleir G. Joye S. B. (2005). Aerobic methane oxidation and methanotroph community composition during seasonal stratification in Mono Lake California (USA). *Environ. Microbiol.* 7 1127–1138. 10.1111/j.1462-2920.2005.00786.x 16011750

[B15] Carpenter S. R. Caraco N. F. Correll D. L. Howarth R. W. Sharpley A. N. Smith V. H. (1998). Nonpoint pollution of surface waters with phosphorus and nitrogen. *Ecol. Appl.* 8 559–568. 10.1890/1051-0761(1998)008[0559:nposww]2.0.co;2

[B16] Casper S. J. (2012). *Lake Stechlin: A Temperate Oligotrophic Lake.* Berlin: Springer Science & Business Media.

[B17] Catherine A. Selma M. Mouillot D. Troussellier M. Bernard C. (2016). Patterns and multi-scale drivers of phytoplankton species richness in temperate peri-urban lakes. *Sci. Total Environ.* 559 74–83. 10.1016/j.scitotenv.2016.03.179 27054495

[B18] Catherine A. Troussellier M. Bernard C. (2008). Design and application of a stratified sampling strategy to study the regional distribution of cyanobacteria (Ile-de-France, France). *Water Res.* 42 4989–5001. 10.1016/j.watres.2008.09.028 18945472

[B19] Chen W. Wilkes G. Khan I. U.-H. Pintar K. D. M. Thomas J. L. Lévesque C. A. (2018). Aquatic bacterial community associated with land use and environmental factors in agricultural landscapes using a metabarcoding approach. *Front. Microbiol.* 9:2301. 10.3389/fmicb.2018.02301 30425684 PMC6218688

[B20] Cires S. Ballot A. (2016). A review of the phylogeny, ecology and toxin production of bloom-forming *Aphanizomenon* spp. and related species within the Nostocales (cyanobacteria). *Harmful Algae* 54 21–43. 10.1016/j.hal.2015.09.007 28073477

[B21] Crump B. C. Adams H. E. Hobbie J. E. Kling G. W. (2007). Biogeography of bacterioplankton in lakes and streams of an arctic tundra catchment. *Ecology* 88 1365–1378. 10.1890/06-0387 17601129

[B22] Crump B. C. Amaral-Zettler L. A. Kling G. W. (2012). Microbial diversity in arctic freshwaters is structured by inoculation of microbes from soils. *ISME J.* 6:1629. 10.1038/ismej.2012.9 22378536 PMC3498914

[B23] Crump B. C. Hopkinson C. S. Sogin M. L. Hobbie J. E. (2004). Microbial biogeography along an estuarine salinity gradient: combined influences of bacterial growth and residence time. *Appl. Environ. Microbiol.* 70 1494–1505. 10.1128/aem.70.3.1494-1505.2004 15006771 PMC365029

[B24] Davis T. W. Bullerjahn G. S. Tuttle T. McKay R. M. Watson S. B. (2015). Effects of increasing nitrogen and phosphorus concentrations on phytoplankton community growth and toxicity during planktothrix Blooms in Sandusky Bay Lake Erie. *Environ. Sci. Technol.* 49 7197–7207. 10.1021/acs.est.5b00799 25992592

[B25] Eiler A. Bertilsson S. (2004). Composition of freshwater bacterial communities associated with cyanobacterial blooms in four Swedish lakes. *Environ. Microbiol.* 6 1228–1243. 10.1111/j.1462-2920.2004.00657.x 15560821

[B26] Fukushima M. Tomioka N. Jutagate T. Hiroki M. Murata T. Preecha C. (2017). The dynamics of pico-sized and bloom-forming cyanobacteria in large water bodies in the Mekong River Basin. *PLoS One* 12:e0189609. 10.1371/journal.pone.0189609 29272288 PMC5741221

[B27] Ghai R. Mizuno C. M. Picazo A. Camacho A. Rodriguez-Valera F. (2014). Key roles for freshwater Actinobacteria revealed by deep metagenomic sequencing. *Mol. Ecol.* 23 6073–6090. 10.1111/mec.12985 25355242

[B28] Glöckner F. O. Zaichikov E. Belkova N. Denissova L. Pernthaler J. Pernthaler A. (2000). Comparative 16S rRNA analysis of lake bacterioplankton reveals globally distributed phylogenetic clusters including an abundant group of actinobacteria. *Appl. Environ. Microbiol.* 66 5053–5065. 10.1128/aem.66.11.5053-5065.2000 11055963 PMC92419

[B29] Grasshoff K. Ehrhardt M. (1999). *Methods Of Seawater Analysis.* 3rd Edn Weinheim: WILEY-VCH, 203–223.

[B30] Grasshoff K. Kremling K. Ehrhardt M. (2009). *Methods of Seawater Analysis.* Hoboken, NJ: John Wiley & Sons.

[B31] Grasshoff K. E. Kremling M. (1983). Methods of seawater analysis. *Verlag Chemie* 4:9.

[B32] Griffin J. S. Lu N. Sangwan N. Li A. Dsouza M. Stumpf A. J. (2017). Microbial diversity in an intensively managed landscape is structured by landscape connectivity. *FEMS Microbiol. Ecol.* 93:fix120. 10.1093/femsec/fix120 28961974

[B33] Hargreaves S. K. Williams R. J. Hofmockel K. S. (2015). Environmental filtering of microbial communities in agricultural soil shifts with crop growth. *PLoS One* 10:e0134345. 10.1371/journal.pone.0134345 26226508 PMC4520589

[B34] Haukka K. Kolmonen E. Hyder R. Hietala J. Vakkilainen K. Kairesalo T. (2006). Effect of nutrient loading on bacterioplankton community composition in Lake Mesocosms. *Microb. Ecol.* 51 137–146. 10.1007/s00248-005-0049-7 16435168

[B35] Hernandez-Agreda A. Gates R. D. Ainsworth T. D. (2017). Defining the core microbiome in corals’ microbial soup. *Trends Microbiol.* 25 125–140. 10.1016/j.tim.2016.11.003 27919551

[B36] Horner-Devine M. C. Lage M. Hughes J. B. Bohannan B. J. M. (2004). A taxa–area relationship for bacteria. *Nature* 432:750. 10.1038/nature03073 15592412

[B37] Hu S. Zeng R. J. Haroon M. F. Keller J. Lant P. A. Tyson G. W. (2015). A laboratory investigation of interactions between denitrifying anaerobic methane oxidation (DAMO) and anammox processes in anoxic environments. *Sci. Rep.* 5:8706. 10.1038/srep08706 25732131 PMC4346804

[B38] Humayoun S. B. Bano N. Hollibaugh J. T. (2003). Depth distribution of microbial diversity in Mono lake, a meromictic Soda Lake in California. *Appl. Environ. Microbiol.* 69 1030–1042. 10.1128/aem.69.2.1030-1042.2003 12571026 PMC143613

[B39] Janssen P. H. (2006). Identifying the dominant soil bacterial Taxa in libraries of 16S rRNA and 16S rRNA genes. *Appl. Environ. Microbiol.* 72 1719–1728. 10.1128/aem.72.3.1719-1728.2006 16517615 PMC1393246

[B40] Joerin F. Thériault M. Musy A. (2001). Using GIS and outranking multicriteria analysis for land-use suitability assessment. *Int. J. Geogr. Inform. Sci.* 15 153–174. 10.1080/13658810051030487

[B41] Jones S. E. McMahon K. D. (2009). Species-sorting may explain an apparent minimal effect of immigration on freshwater bacterial community dynamics. *Environ. Microbiol.* 11 905–913. 10.1111/j.1462-2920.2008.01814.x 19040451

[B42] Katsiapi M. Mazaris A. D. Charalampous E. Moustaka-Gouni M. (2012). Watershed land use types as drivers of freshwater phytoplankton structure. *Hydrobiologia* 698 121–131. 10.1007/978-94-007-5790-5_10

[B43] Khadem A. F. Pol A. Jetten M. S. M. Op den Camp H. J. M. (2010). Nitrogen fixation by the verrucomicrobial methanotroph ‘*Methylacidiphilum fumariolicum*’ SolV. *Microbiology* 156 1052–1059. 10.1099/mic.0.036061-0 20056702

[B44] King A. J. Freeman K. R. McCormick K. F. Lynch R. C. Lozupone C. Knight R. (2010). Biogeography and habitat modelling of high-alpine bacteria. *Nat. Commun.* 1:53. 10.1038/ncomms1055 20975720

[B45] Kolmonen E. Sivonen K. Rapala J. Haukka K. (2004). Diversity of cyanobacteria and heterotrophic bacteria in cyanobacterial blooms in Lake Joutikas Finland. *Aqua. Microb. Ecol.* 36 201–211. 10.3354/ame036201

[B46] Kopylov A. I. Kosolapov D. B. Romanenko A. V. Degermendzhy A. G. (2002). Structure of planktonic microbial food web in a brackish stratified Siberian lake. *Aqua. Ecol.* 36 179–204.

[B47] Kozak A. Celewicz-Gołdyn S. Kuczyńska-Kippen N. (2019). Cyanobacteria in small water bodies: the effect of habitat and catchment area conditions. *Sci. Total Environ.* 646 1578–1587. 10.1016/j.scitotenv.2018.07.330 30235642

[B48] Langenheder S. Székely A. J. (2011). Species sorting and neutral processes are both important during the initial assembly of bacterial communities. *ISME J.* 5 1086–1094. 10.1038/ismej.2010.207 21270841 PMC3146284

[B49] Larned S. T. Scarsbrook M. R. Snelder T. H. Norton N. J. Biggs B. J. F. (2004). Water quality in low-elevation streams and rivers of New Zealand: recent state and trends in contrasting land-cover classes. *New Zealand J. Mar. Freshw. Res.* 38 347–366. 10.1080/00288330.2004.9517243

[B50] Lear G. Washington V. Neale M. Case B. Buckley H. Lewis G. (2013). The biogeography of stream bacteria. *Glob. Ecol. Biogeogr.* 22 544–554. 10.1111/geb.12046

[B51] Leibold M. A. Norberg J. (2004). Biodiversity in metacommunities: plankton as complex adaptive systems? *Limnol. Oceanogr.* 49 1278–1289. 10.4319/lo.2004.49.4_part_2.1278

[B52] Lindström E. S. Bergström A.-K. (2004). Influence of inlet bacteria on bacterioplankton assemblage composition in lakes of different hydraulic retention time. *Limnol. Oceanogr.* 49 125–136. 10.4319/lo.2004.49.1.0125

[B53] Lindström E. S. Kamst-Van Agterveld M. P. Zwart G. (2005). Distribution of typical freshwater bacterial groups is associated with pH, temperature, and Lake water retention time. *Appl. Environ. Microbiol.* 71 8201–8206. 10.1128/aem.71.12.8201-8206.2005 16332803 PMC1317352

[B54] Lindström E. S. Langenheder S. (2012). Local and regional factors influencing bacterial community assembly. *Environ. Microbiol. Rep.* 4 1–9. 10.1111/j.1758-2229.2011.00257.x 23757223

[B55] Logares R. Tesson S. V. Canbäck B. Pontarp M. Hedlund K. Rengefors K. (2018). Contrasting prevalence of selection and drift in the community structuring of bacteria and microbial eukaryotes. *Environ. Microbiol.* 20 2231–2240. 10.1111/1462-2920.14265 29727053

[B56] Logue J. B. Langenheder S. Andersson A. F. Bertilsson S. Drakare S. Lanzén A. (2012). Freshwater bacterioplankton richness in oligotrophic lakes depends on nutrient availability rather than on species–area relationships. *ISME J.* 6:1127. 10.1038/ismej.2011.184 22170419 PMC3358030

[B57] Logue J. B. Lindström E. S. (2008). Biogeography of bacterioplankton in inland waters. *Freshw. Rev.* 1 99–115.

[B58] Logue J. B. Lindström E. S. (2010). Species sorting affects bacterioplankton community composition as determined by 16S rDNA and 16S rRNA fingerprints. *ISME J.* 4:729. 10.1038/ismej.2009.156 20130658

[B59] Logue J. B. Stedmon C. A. Kellerman A. M. Nielsen N. J. Andersson A. F. Laudon H. (2015). Experimental insights into the importance of aquatic bacterial community composition to the degradation of dissolved organic matter. *ISME J.* 10:533. 10.1038/ismej.2015.131 26296065 PMC4817675

[B60] Louca S. Parfrey L. W. Doebeli M. (2016). Decoupling function and taxonomy in the global ocean microbiome. *Science* 353 1272–1277. 10.1126/science.aaf4507 27634532

[B61] Mantzouki E. Lürling M. Fastner J. de Senerpont Domis L. Wilk-Woźniak E. Koreivienë J. (2018). Temperature effects explain continental scale distribution of cyanobacterial toxins. *Toxins* 10:156. 10.3390/toxins10040156 29652856 PMC5923322

[B62] Marcelino V. R. van Oppen M. J. H. Verbruggen H. (2017). Highly structured prokaryote communities exist within the skeleton of coral colonies. *ISME J.* 12:300. 10.1038/ismej.2017.164 29053151 PMC5739017

[B63] Marmen S. Aharonovich D. Grossowicz M. Blank L. Yacobi Y. Z. Sher D. J. (2016). Distribution and Habitat specificity of potentially-toxic microcystis across climate, land, and water use gradients. *Front. Microbiol.* 7:271. 10.3389/fmicb.2016.00271 27014200 PMC4791393

[B64] Marmen S. Blank L. Al-Ashhab A. Malik A. Ganzert L. Lalzar M. (2018). The role of land use types and water chemical properties in structuring the microbiome of a connected lakes system. *bioRxiv [Preptint]* 10.1101/401299 PMC7029742 32117119

[B65] Mašín M. Jezbera J. Nedoma J. Straškrabová V. Hejzlar J. Šimek K. (2003). Changes in bacterial community composition and microbial activities along the longitudinal axis of two canyon-shaped reservoirs with different inflow loading. *Hydrobiologia* 504 99–113. 10.1023/b:hydr.0000008512.04563.0b

[B66] Mehaffey M. H. Nash M. S. Wade T. G. Ebert D. W. Jones K. B. Rager A. (2005). Linking land cover and water quality in New York City’S water supply watersheds. *Environ. Monitor. Assess.* 107 29–44. 10.1007/s10661-005-2018-5 16418903

[B67] Mitsui A. Kumazawa S. Takahashi A. Ikemoto H. Cao S. Arai T. (1986). Strategy by which nitrogen-fixing unicellular cyanobacteria grow photoautotrophically. *Nature* 323:720 10.1038/323720a0

[B68] Ndlela L. L. Oberholster P. J. Van Wyk J. H. Cheng P. H. (2016). An overview of cyanobacterial bloom occurrences and research in Africa over the last decade. *Harmful Algae* 60 11–26. 10.1016/j.hal.2016.10.001 28073554

[B69] Nekola J. C. White P. S. (1999). The distance decay of similarity in biogeography and ecology. *J. Biogeogr.* 26 867–878. 10.1046/j.1365-2699.1999.00305.x

[B70] Nelson C. E. Sadro S. Melack J. M. (2009). Contrasting the influences of stream inputs and landscape position on bacterioplankton community structure and dissolved organic matter composition in high-elevation lake chains. *Limnol. Oceanogr.* 54 1292–1305. 10.4319/lo.2009.54.4.1292

[B71] Newton R. J. Jones S. E. Eiler A. McMahon K. D. Bertilsson S. (2011). A guide to the natural history of freshwater lake bacteria. *Microbiol. Mol. Biol. Rev.* 75 14–49. 10.1128/MMBR.00028-10 21372319 PMC3063352

[B72] Niño-García J. P. Ruiz-González C. del Giorgio P. A. (2016). Interactions between hydrology and water chemistry shape bacterioplankton biogeography across boreal freshwater networks. *ISME J.* 10:1755. 10.1038/ismej.2015.226 26849312 PMC4918434

[B73] Nogueira R. Melo L. F. Purkhold U. Wuertz S. Wagner M. (2002). Nitrifying and heterotrophic population dynamics in biofilm reactors: effects of hydraulic retention time and the presence of organic carbon. *Water Res.* 36 469–481. 10.1016/s0043-1354(01)00229-9 11827353

[B74] Op den Camp H. J. Islam T. Stott M. B. Harhangi H. R. Hynes A. Schouten S. (2009). Environmental, genomic and taxonomic perspectives on methanotrophic Verrucomicrobia. *Environ. Microbiol. Rep.* 1 293–306. 10.1111/j.1758-2229.2009.00022.x 23765882

[B75] Percent S. F. Frischer M. E. Vescio P. A. Duffy E. B. Milano V. McLellan M. (2008). Bacterial community structure of acid-impacted lakes: what controls diversity? *Appl. Environ. Microbiol.* 74 1856–1868. 10.1128/AEM.01719-07 18245245 PMC2268290

[B76] Proctor C. R. Besmer M. D. Langenegger T. Beck K. Walser J. C. Ackermann M. (2018). Phylogenetic clustering of small low nucleic acid-content bacteria across diverse freshwater ecosystems. *ISME J.* 12 1344–1359. 10.1038/s41396-018-0070-8 29416124 PMC5932017

[B77] Quinn J. M. Cooper A. B. Davies-Colley R. J. Rutherford J. C. Williamson R. B. (1997). Land use effects on habitat, water quality, periphyton, and benthic invertebrates in Waikato, New Zealand, hill-country streams. *New Zealand J. Mar. Freshw. Res.* 31 579–597. 10.1080/00288330.1997.9516791

[B78] Reimann C. Finne T. E. Nordgulen Ø Sæther O. M. Arnoldussen A. Banks D. (2009). The influence of geology and land-use on inorganic stream water quality in the Oslo region, Norway. *Appl. Geochem.* 24 1862–1874. 10.1016/j.apgeochem.2009.06.007

[B79] Ritchie R. J. (2008). Universal chlorophyll equations for estimating chlorophylls a, b, c, and d and total chlorophylls in natural assemblages of photosynthetic organisms using acetone, methanol, or ethanol solvents. *Photosynthetica* 46 115–126. 10.1007/s11099-008-0019-7

[B80] Savage C. Leavitt P. R. Elmgren R. (2010). Effects of land use, urbanization, and climate variability on coastal eutrophication in the Baltic Sea. *Limnol. Oceanogr.* 55 1033–1046. 10.4319/lo.2010.55.3.1033

[B81] Savio D. Sinclair L. Ijaz U. Z. Parajka J. Reischer G. H. Stadler P. (2015). Bacterial diversity along a 2600 km river continuum. *Environ. Microbiol.* 17 4994–5007. 10.1111/1462-2920.12886 25922985 PMC4918796

[B82] Schoonover J. E. Lockaby B. G. (2006). Land cover impacts on stream nutrients and fecal coliform in the lower Piedmont of West Georgia. *J. Hydrol.* 331 371–382. 10.1016/j.jhydrol.2006.05.031

[B83] Shade A. Handelsman J. (2011). Beyond the Venn diagram: the hunt for a core microbiome. *Environ. Microbiol.* 14 4–12. 10.1111/j.1462-2920.2011.02585.x 22004523

[B84] Singer E. Bushnell B. Coleman-Derr D. Bowman B. Bowers R. M. Levy A. (2016). High-resolution phylogenetic microbial community profiling. *ISME J.* 10:2020. 10.1038/ismej.2015.249 26859772 PMC5029162

[B85] Solomon C. T. Jones S. E. Weidel B. C. Buffam I. Fork M. L. Karlsson J. (2015). Ecosystem consequences of changing inputs of terrestrial dissolved organic matter to lakes: current knowledge and future challenges. *Ecosystems* 18 376–389. 10.1007/s10021-015-9848-y

[B86] Sukenik A. Hadas O. Kaplan A. Quesada A. (2012). Invasion of Nostocales (cyanobacteria) to subtropical and temperate freshwater lakes – physiological, regional, and global driving forces. *Front. Microbiol.* 3:86. 10.3389/fmicb.2012.00086 22408640 PMC3297820

[B87] Theobald T. F. Schipper M. Kern J. (2016). Phosphorus flows in Berlin-Brandenburg, a regional flow analysis. *Resour. Conserv. Recycl.* 112 1–14. 10.1016/j.resconrec.2016.04.008

[B88] Valério E. Chaves S. Tenreiro R. (2010). Diversity and impact of prokaryotic toxins on aquatic environments: a review. *Toxins* 2:2359. 10.3390/toxins2102359 22069558 PMC3153167

[B89] Van der Gucht K. Cottenie K. Muylaert K. Vloemans N. Cousin S. Declerck S. (2007). The power of species sorting: local factors drive bacterial community composition over a wide range of spatial scales. *Proc. Natl. Acad. Sci. U.S.A.* 104 20404–20409. 10.1073/pnas.0707200104 18077371 PMC2154443

[B90] Van Rossum T. Peabody M. A. Uyaguari-Diaz M. I. Cronin K. I. Chan M. Slobodan J. R. (2015). Year-long Metagenomic study of river microbiomes across land use and water quality. *Front. Microbiol.* 6:1405. 10.3389/fmicb.2015.01405 26733955 PMC4681185

[B91] Vitousek P. M. Mooney H. A. Lubchenco J. Melillo J. M. (1997). Human domination of Earth’s ecosystems. *Science* 277 494–499. 10.1126/science.277.5325.494

[B92] Wang X. Yin Z.-Y. (1997). Using GIS to assess the relationship between land use and water quality at a watershed level. *Environ. Int.* 23 103–114. 10.1016/s0160-4120(96)00081-5 17106781

[B93] Wei Y. Wei Z. Cao Z. Zhao Y. Zhao X. Lu Q. (2016). A regulating method for the distribution of phosphorus fractions based on environmental parameters related to the key phosphate-solubilizing bacteria during composting. *Bioresour. Technol.* 211 610–617. 10.1016/j.biortech.2016.03.141 27043056

[B94] Williamson C. E. Saros J. E. Vincent W. F. Smol J. P. (2009). Lakes and reservoirs as sentinels, integrators, and regulators of climate change. *Limnol. Oceanogr.* 54 2273–2282. 10.1016/j.scitotenv.2011.07.069 21962562

[B95] Winter T. C. Rosenberry D. O. LaBaugh J. W. (2003). Where does the ground water in small watersheds come from? *Groundwater* 41 989–1000. 10.1111/j.1745-6584.2003.tb02440.x

[B96] Wolz C. R. M. Yarwood S. A. Grant E. H. C. Fleischer R. C. Lips K. R. Hoye B. (2018). Effects of host species and environment on the skin microbiome of Plethodontid salamanders. *J. Anim. Ecol.* 87 341–353. 10.1111/1365-2656.12726 28682480

[B97] Xu H. Paerl H. W. Qin B. Zhu G. Gaoa G. (2010). Nitrogen and phosphorus inputs control phytoplankton growth in eutrophic Lake Taihu China. *Limnol. Oceanogr.* 55 420–432. 10.4319/lo.2010.55.1.0420

[B98] Yannarell A. C. Triplett E. W. (2005). Geographic and environmental sources of variation in lake bacterial community composition. *Appl. Environ. Microbiol.* 71 227–239. 10.1128/aem.71.1.227-239.2005 15640192 PMC544217

[B99] Zeglin L. H. (2015). Stream microbial diversity in response to environmental changes: review and synthesis of existing research. *Front. Microbiol.* 6:454. 10.3389/fmicb.2015.00454 26042102 PMC4435045

[B100] Zhang J. Su Y. Wu J. Liang H. (2015). GIS based land suitability assessment for tobacco production using AHP and fuzzy set in Shandong province of China. *Comput. Electr. Agric.* 114 202–211. 10.1016/j.compag.2015.04.004

