# Supplementary material for: The Role of Land Use Types and Water Chemical Properties in Structuring the Microbiomes of a Connected Lake System
Source: Front Microbiol. 2020 Feb 12;11:89. doi: 10.3389/fmicb.2020.00089 (PMC7029742; doi:10.3389/fmicb.2020.00089)
Supplement: Supplementary file 1 [file Data_Sheet_1.docx]

Supplementary Material

**The role of land use types and water chemical properties in structuring the microbiomes of a connected lake system**

Sophi Marmen^1^, Lior Blank^2^, Ashraf Al-Ashhab^1,3^, Assaf Malik^4^, Lars Ganzert^5^, Maya Lalzar^4^, Hans-Peter Grossart^5,5^ and Daniel Sher^1*^

^1^ *Department of Marine Biology, Leon H. Charney School of Marine Sciences, University of Haifa, Israel*

*^2^ Department of Plant Pathology and Weed Research, ARO, Volcani Center, Rishon Lezion, Israel*

*^3^ Microbial Metagenomics Division, Dead Sea and Arava Science Center, Masada, Israel*

*^4^ Bioinformatics Service Unit, University of Haifa, Israel*

*^5^ Leibnitz Institute for Freshwater Ecology and Inland Fisheries, Department of Experimental Limnology, Stechlin, Germany*

*^6^ Potsdam University, Institute of Bicochemistry and Biology, Maulbeerallee 2, 24469 Potsdam, Germany*

^*^Corresponding author: Dr. Daniel Sher, Department of Marine Biology, Leon H. Charney School of Marine Sciences, University of Haifa, Haifa 31905, Israel. email: [dsher@univ.haifa.ac.il](mailto:dsher@univ.haifa.ac.il)

**1. Supplementary methods**

**1.1 Study region and delineation of drainage basins**

The sampling area lies within the region of Brandenburg, in northern Germany (Figure 1, Supplementary Figure 1). The area is relatively flat (18-89 meters above sea level) and contains hundreds of lakes, ponds and depressions that were formed by glacial action during the Pleistocene. Three main basins drain this region. From west to east, these are: 1) The Rhin basin, which flows south and then south-east, joining the Havel basin near Oranienburg; 2) The Havel basin itself, which flows generally south from the area of Lake Müritz, joining the Lychen sub-basin (which flows from the east) at Stolpsee (nearby Fürstenberg). It then continues south through Oranienburg to Berlin. The Havel then turns west and drains into the Elbe, itself draining into the North Sea, northwest of Hamburg; 3) the Ucker basin, flowing north through Oberuckersee and Unteruckersee and finally draining into the Szczecin Lagoon and the Baltic Sea. These three main basins can be subdivided into smaller basins.

The drainage basin of each lake was defined as the line connecting the highest points from which the surface water flow enters each of the lakes. Since the lakes are inter-connected, the drainage basin of each lake contains also those of the lakes upstream to it. Due to the flat topography of the region automatic basin detection using GIS was not satisfactory, and thus the basins depicted in Figure 1 and Supplementary Figure 1 were delineated manually using a combination of topographic maps produced from the Digital Elevation Model layer with a resolution of 0.0008333 decimal degree and high-resolution hiking and biking maps (Kompass Fahrradkarte, 1:70,000). Human structures, such as roads were not informative due to the flat terrain. Literature surveys and site visits by the sampling team were used to clarify connectivity and flow conditions at specific locations. Notably, due to the flat terrain and the long history of water use for agriculture, aquaculture and transportation, the region includes also many artificial canals, weirs and ponds, often connecting between different basins. In order to simplify the analysis, the exchange between the basins was neglected. Additionally, Lake Müritz primarily drains north-east into Lake Kölpinsee and from there east into the Elde and ultimately into the Elbe. However, the lake also drains south at Rechlin into the Havel basin. The border between the Havel and Elde basins was delineated, based on the topography surrounding the lake, as shown in Figure 1.

Our 46 sampling sites varied between 7 different sub-basins as follow: 11 samples at Havel basin, 10 at Lychen, 12 at Rhin, 5 at Stechlin, 4 at Ucker, 1 sample from Tollensesee, 1 sample from Berlin Spree and 2 samples from lakes that are not part from any drainage basin (marked as ”none” in Figure 1). Not all samples from the same basin are directly connected, for instance, water from the lakes in the Rhin’s northern part (12-15 in Supplementary Figure 1) meets water from the lakes in the Rhin’s eastern part (18, 19, 21-23 in Supplementary Figure 1) only downstream, near sites 16 and 17. Thus, in some cases sub-basins should be considered for the analyses (see main text and Sup Supplementary Figure 5).

The land uses, obtained from the Corine Land Cover map (CLC, <http://www.eea.europa.eu/publications/COR0-landcover>), were divided into five categories: Artificial areas, Agriculture (further divided for GLM analysis to heterogeneous agriculture, pasture lands and arable lands), Forests and natural areas, Wetlands and Water bodies (Figure 1). Sample locations were selected in order to represent a wide diversity of environmental parameters and land uses (Supplementary Data File 1, Supplementary Figure 1).

**1.2 Estimation of the trophic status of the lakes**

The trophic classification of the lakes is based on measurements done or directed by the environmental agencies of the federal states Mecklenburg-Vorpommern and Brandenburg, following in general the rules of the water framework directive of the European Union. Such assessments are available for 25 out of the 46 lakes in the following link https://lfu.brandenburg.de/cms/detail.php/bb1.c.305410.de

Information for another 14 lakes was gathered from the following additional websites:

https://www-docs.b-tu.de/fg-gewaesserschutz/public/projekte/uba_2/02_meck_pom.pdf

https://www-docs.b-tu.de/fg-gewaesserschutz/public/projekte/uba_2/05_brandenburg.pdf

https://www.lung.mv-regierung.de/dateien/glrp_ms_06_2011.pdf

Finally, the trophic state of the rest of the lakes was assessed based on the local expertise of the authors. Based on these assessments, there was one meso-oligotrophic lake, 8 mesotrophic lakes, 30 eutrophic lakes and 7 polytrophic lakes (including one fishpond, Supplementary excel file).

**1.3 DNA extraction, sequencing of 16S rRNA gene amplicons and sequence quality control**

DNA extraction was performed at the Biomedical Core Facility of the Technion, Israel, using a combination of manual treatments and robotic extraction with the QiaCube robot. Briefly, filters were thawed, centrifuged for 10 min at 15000 × g and the preservation buffer was removed. Lysis buffer from the DNeasy Blood & Tissue Kit (Qiagen) was added and the samples were mechanically pounded with two 3mm sterile stainless steel beads at the speed of 30 Hz for 1.5 min using a TissueLyser LT (Qiagen). 30µl of lysozyme were added, the tubes were incubated at 37^o^C for 30 min, followed by the addition of 25mL proteinase K and 200μl of the buffer AL with an additional incubation for 1 h at 56°C on a shaker. Finally, the tubes were centrifuged for 10 min at 5000 x *g* and the upper liquid was transferred to a new 2mL Eppendorf tube for extraction by the QiaCube robot using the manufacturer’s instructions. The DNA quantity was assessed using Picogreen (Quant-iT PicoGreen dsDNA reagent, Invitrogen), and the quality (apparent size and degradation products) was assessed using a TapeStation (Agilent Technologies).

PCR reactions for amplifying the 16S rRNA gene fragments were performed in triplicate for each DNA sample using the following primers targeting the V3-V4 region: forward CS1_341F (5’-ACA CTG ACG ACA TGG TTC TAC ANN NNC CTA CGG GAG GCA GCA G-3’) and reverse CS2_806R (5’-TAC GGT AGC AGA GAC TTG GTC TGG ACT ACH VGG GTW TCT AAT-3’). The PCR reactions were performed in a volume of 25μl, with 10ng of DNA as PCR template. The PCR protocol was as follows: initial denaturation stage at 95°C for 5 min, followed by 28 cycles at 95°C for 30s, 50°C for 30s, and 72°C for 60s, with a final extension step at 72°C for 5 min. The enzyme used was BIOLINE 2x MyTaq Red Mix, and the PCR reaction was performed in a TProfessional Basic Gradient thermocycler (Biometra). In each PCR, negative controls in a form of distilled water, instead of DNA, were added, and did not produced any bands in the gels. Following the first PCR, triplicate samples were combined, and the products sent to the DNA Services Facility of the University of Illinois, Chicago, where a second PCR was performed to incorporate barcodes and sequencing adapters (CS1 and CS2). Sequencing was performed using a 2×250 base pair format on a MiSeq flow cell (V3 chemistry). No cross contamination was observed between the freshwater samples described here, human samples and samples from axenic cultures of *Prochlorococccus*, ruling out cross contamination during the sequencing stages.

One control unfortunately lacking from our study is blank filtration controls, where pure water was collected in the same manner as the lake samples and filtered similarly. Therefore, while we cannot rule out completely any chance of cross contamination during the filtration stage, such cross contamination likely had a very limited effect on our results. First, we tested the efficiency of our cleaning protocol on a sample of seawater, counting the number of phytoplankton cells remaining after the washing procedure using flow cytometry. The washing procedure reduced the number of cells counted by flow cytometry from 17,726 cell/ml to less than 250 cells/ml (at least 50-fold, to within the range of background instrument noise). Second, we removed all OTUs with fewer than 5 reads, as the presence of such ”very rare” organisms could be due to multiple reasons, including cross-contamination but also PCR errors etc. Thus, we can expect cross contamination to have a negligible effect on the relative abundances of the more-common organisms, and our analyses do not focus on the rare organisms. We note that the use of bleach to remove contaminating DNA between samples is recommended (e.g. for filtration devices), yet is not always feasible – e.g. oceanographic cruises do not wash the Niskin bottles with bleach between casts. We also note that there are multiple additional sources of contamination in microbiome studies, including the filters and the kits and reagents used for PCR amplification (Salter et al., 2014), and these aspects should be taken into account. Of the list of common contaminating bacteria presented by Salter and co-workers, only Novosphingobium was present at an average abundance of more than 0.1% of the population, and this clade includes aquatic organisms isolated from freshwater, estuarine and marine environments, thus likely representing a bona-fide member of the microbial community in the sampled lakes.

**1.4 16S analysis and statistical tests**

Upon receiving the sequence data all sequences underwent quality control for Phix DNA removal using bowtie2 (Langmead & Salzberg, 2012), unmerged reads using PEAR (Zhang*, et al.*, 2013) and incomplete, low quality scores and ambiguous bases sequences using MOTHUR Software V.1.36.1 using a phred score of 30, removing homopolymers longer than 8 nucleotides long and allowing no ambiguous bases (Schloss*, et al.*, 2009). Following quality filtering, sequences were classified into different Operational Taxonomic Units (OTUs) based on 97% sequence similarity threshold using the “pick_de_novo_otus.py” command in Qiime (Caporaso*, et al.*, 2010) and assigned taxonomy with the SILVA database version 128. Upon classification, an OTU table was generated and OTUs with fewer than 5 reads across the entire table were removed. At the end of analysis, the range of sequence number per sample was 35,775 – 75,704. This dataset, containing 45,240 OTUs, was used for all diversity analyses, including Bray – Curtis dissimilarity calculations and nMDS analysis (Supplementary figure Fig. 3, 5). For all multivariate statistical analyses and for the graphs presented in Figure 2B we used a smaller dataset which included only 3,611 OTUs with at least 50 reads across all sites. OTU numbers were log_10_ transformed. All calculations were performed in R software version 3.3.3 using the vegan package (Oksanen*, et al.*, 2007).

Alpha diversity (Shannon index) was calculated using the ”diversity” function. The similarity in bacterial community structure among samples was calculated using a Bray–Curtis dissimilarity by ”vegdist” function. Gamma diversity was calculated in Excel by summing the number of sites where each OTU was present and dividing them into five categories: 1) Core OTUs, which were present at all the locations; 2) Cosmopolitan OTUs, which were present at more than 90% of the locations; 3) Common OTUS, present at more than 50% of the sites; 4) Rare OTUs, present in less than 10% of the sites; and 5) Unique OTUs, specific to only one site.

In order to explore whether samples from the same drainage basin have spatial similarity of bacterial communities, a non-metric multidimensional scaling analysis (nMDS) was carried out in R with ”metaMDS” function and a Bray-Curtis matrix. Sites were colored by basins.

Before performing multivariate analyses, we removed environmental parameters which were strongly correlated (Spearman *r* > 0.8, *P* < 0.05), with the exception of forests and arable lands, as the removal of one of these dominant land-use types significantly reduced the total fractional cover. Thus, the effect of these land-use types cannot be differentiated. The final environmental parameters selected were pH, temperature, ammonium, NO_2_ + NO_3_ total phosphorus and *Chl a*, generally referred as ”aquatic environmental parameters”. Variation partitioning analysis (VPA, Legendre*, et al.*, 2012) was used to describe the partitioning of variation in water microbiome among three data sets: ”Local Land Use” (the fractional land use within 500 m of the water body) and ”Basin Land Use” (the fractional land use within the drainage basin of each water body) and aquatic environmental parameters, see Supplementary Data File 1, sheet ”metadata”. For the analysis, we included thirteen land use types in the basin data-set and ten in the local (see Supplementary Data File 1). Land use types which were measurable at less in four sites were removed from analysis (e.g. Basin scale: Industrial areas, Construction areas, Artificial land cover, Crops, Vegetation, Open agriculture spaces and Wetlands; Local scale: Industrial areas, Artificial land cover, Herbaceous areas and Wetlands). We note that Forest and Arable lands have a strong negative correlation (Pearson *r* = -0.86), however, since in most basins these two land-use types contributes as much as 70-90% of the total area we did not remove one in order to avoid biases due to different total land-use values. The final table of land uses included six types in both scales: Urban fabric, Heterogenous agriculture, Pastures, Forests, Arable lands and Water bodies (see Supplementary Data File 1).

In order to validate the VPA results and further describe the effects of the different land use types on OTU patters, we performed canonical correspondence analysis (CCA) and permutation test (nperm=1000) using ”cca” and ”anova.cca” commands in R package ”vegan”. We also considered whether the models have significant independent explanatory power by testing conditioned CCA. In the case of testing for model “Basin Land Use”, “Local Land Use” parameters were used as conditioning variables and vice versa.

VPA and CCA were also used to evaluate partitioning of variation in the bacterial community structure with three groups of explanatory variables: ”aquatic environmental parameters”, ”Local Land Use”, and ”Basin Land Use” (Supplementary Data File 1). Since ”varpart” cannot be performed with missing data, we had to remove four sites (i.e. HAR, S.wen, Wen, F.P, Supplementary Data File 1) from the data-set before performing the analysis. Part of the removed samples are located around Berlin, for which we did not have detailed information on the drainage basin, and part were missing aquatic environmental measurements due to technical problems (i.e. Wan, Has and Hol).

We note that VPA analysis (as performed in R using the ”vegan” package) assesses the explained variance by the environmental parameters against the randomly explained variance. That process is performed for each of the matrices and for a combination of matrices, e.g. water chemistry + basin land use, water chemistry + local land use, etc. For each matrix or combination, there are three options for the results:

1. The explained variance by each environmental matrix, or by a combination of matrixes, is higher than the randomly explained variance. In that case a positive value is obtained.

2. The explained variance by each environmental matrix, or by a combination of matrixes, is equal to the randomly explained variance. In that case a zero is obtained.

3. The explained variance by each environmental matrix, or by a combination of matrixes, is lower than the randomly explained variance. In that case a negative value is obtained.

**2. Supplementary Tables**

**Supplementary Table 1:** Statistical values of each of the land uses in the GLMs correlations to water properties. The following abbreviations are used: U – Urban areas, Ar – Heterogenous agriculture, Pas – Pastures lands, Ag – Heterogeneous agriculture, For – Forests, W – Water bodies, *chl* a – *chlorophyll* a, TP – total phosphorus

| Water parameter | Land uses | Level of  land use | Model’s  R^2^ | Main contributing land uses for the correlation |
| --- | --- | --- | --- | --- |
| NO_2_+NO_3_ | U+Ar+Pas+Ag+For+W | Local | 0.17 | Arable (+) |
|  | U+Ar+Pas+Ag+For+W | Basin | 0.74 | Urban (+) |
| pH | U+Ar+Pas+Ag+For+W | Local | 0.13 | ‒ |
|  | U+Ar+Pas+Ag+For+W | Basin | 0.25 | Forests (-) |
| Ammonium | U+Ar+Pas+Ag+For+W | Local | 0.08 | ‒ |
|  | U+Ar+Pas+Ag+For+W | Basin | 0.06 | ‒ |
| P | U+Ar+Pas+Ag+For+W | Local | 0.02 | ‒ |
|  | U+Ar+Pas+Ag+For+W | Basin | 0.14 | ‒ |
| Chl a | U+Ar+Pas+Ag+For+W | Local | 0.06 | ‒ |
|  | U+Ar+Pas+Ag+For+W | Basin | 0.078 | ‒ |

**Supplementary Table 2** Importance values of each of the basin's land uses in the GLMs. Abbreviations: *Chl a* – *chlorophyll* *a*, TP – total phosphorus

|  | Chl a | NO_2_+NO_3_ | pH | Ammonium | TP |
| --- | --- | --- | --- | --- | --- |
| Agriculture area | 0.26 | 0.33 | 0.075 | 0.16 | 0.14 |
| Pastures lands | 0.23 | 0.17 | 0.45 | 0.13 | 0.13 |
| Arable Lands | 0.3 | 0.16 | 0.71 | 0.32 | 0.12 |
| Urban area | 0.24 | 1.00 | 0.14 | 0.14 | 0.12 |
| Forests | 0.36 | 0.19 | 0.19 | 0.3 | 0.12 |
| Water bodies | 0.31 | 0.15 | 0.44 | 0.13 | 0.31 |

**Supplementary Table 3: Spearman correlations between the abundance of specific OTUs and different environmental parameters.** The only significant correlations occur between two OTUs and the water concentrations of NO_2_+NO_3_

| Phylum | Order | Class | Family | Genus | Enviro-nmental parameter | r2 | Adj  p val |
| --- | --- | --- | --- | --- | --- | --- | --- |
| Planctomyce-tes | Phycisphaerae | Phycisphae-rales | NA | NA | NO_2_+NO_3_ | 0.53 | 0 |
| Bacteroide-tes | Saprospir-ae | Saprospiral-es | Saprospira-ceae | Aquirestis | NO_2_+NO_3_ | 0.41 | 0 |

**Supplementary Table 4: VPA and CCA results to describe the partitioning of water microbiome by variation in aquatic environmental parameters and land uses.** Significance of the CCA models tested using Anova. * represents statistical significance.

| **Analysis** |  | Water chemistry | Basin Land Use | Local Land use |
| --- | --- | --- | --- | --- |
| Variation partitioning | Total VE (%) | 11.3* | 5.5 | 1.3 |
|  | Independent VE (%) | 11* | 4.5 | 0 |
|  |  |  |  |  |
| CCA | Inertia explained (%) | 24.2* | 17.6 | 13.9 |
|  | Significant variables (*P*<0.05) | Temperature;  NO_2_+NO_3_  Total P;  *Chl* a |  |  |
|  |  |  |  |  |
| Partial CCA (conditioned) | Inertia explained (%) | 21.4 |  |  |
|  | Significant variables (*P*<0.05) | Total P;  NO_2_+NO_3_ |  |  |

**3. Supplementary Figures**

**
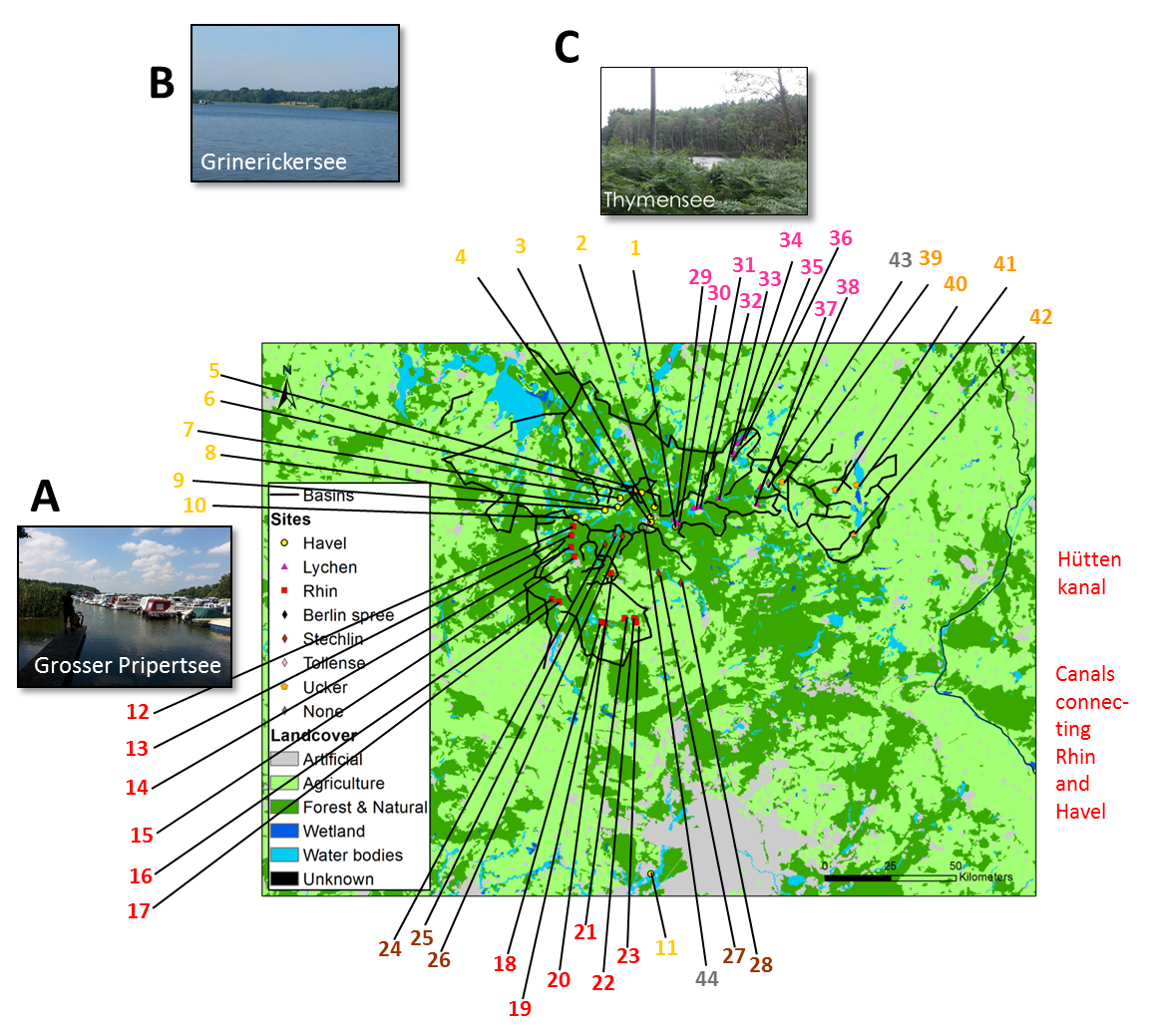
**

**Supplementary Figure 1. Detailed map of the sampling location, with the different sampling sites noted.** Sampling sites were chosen to represent a high diversity in environmental gradients, e.g. variety in land use and water chemistry. These differences between the sites can be visualized - even before our measurement: a-c) Lakes of the Havel basin. a) Grosser Priepertsee is lake located within a town called Priepert and is used for boating and recreation, while b) Grienericksee is located next to the city of Rheinsberg with clear blue water. c) Thymensee is located in the middle of a forest area far away from any town. Arrows with numbers indicate sites as are presented in Supplementary Excel table. Red lines represent connectivity between drainage basins, as mentioned in the text. Notice that two of the sites studied (Hölzerner See and Tollensesee) are outside the scope of this map. The GPS coordinates of the lakes can be found in the Supplementary Excel table under the numbers 45 and 46 (note that these coordinates are of the lakes and not the exact sampling sites).

**
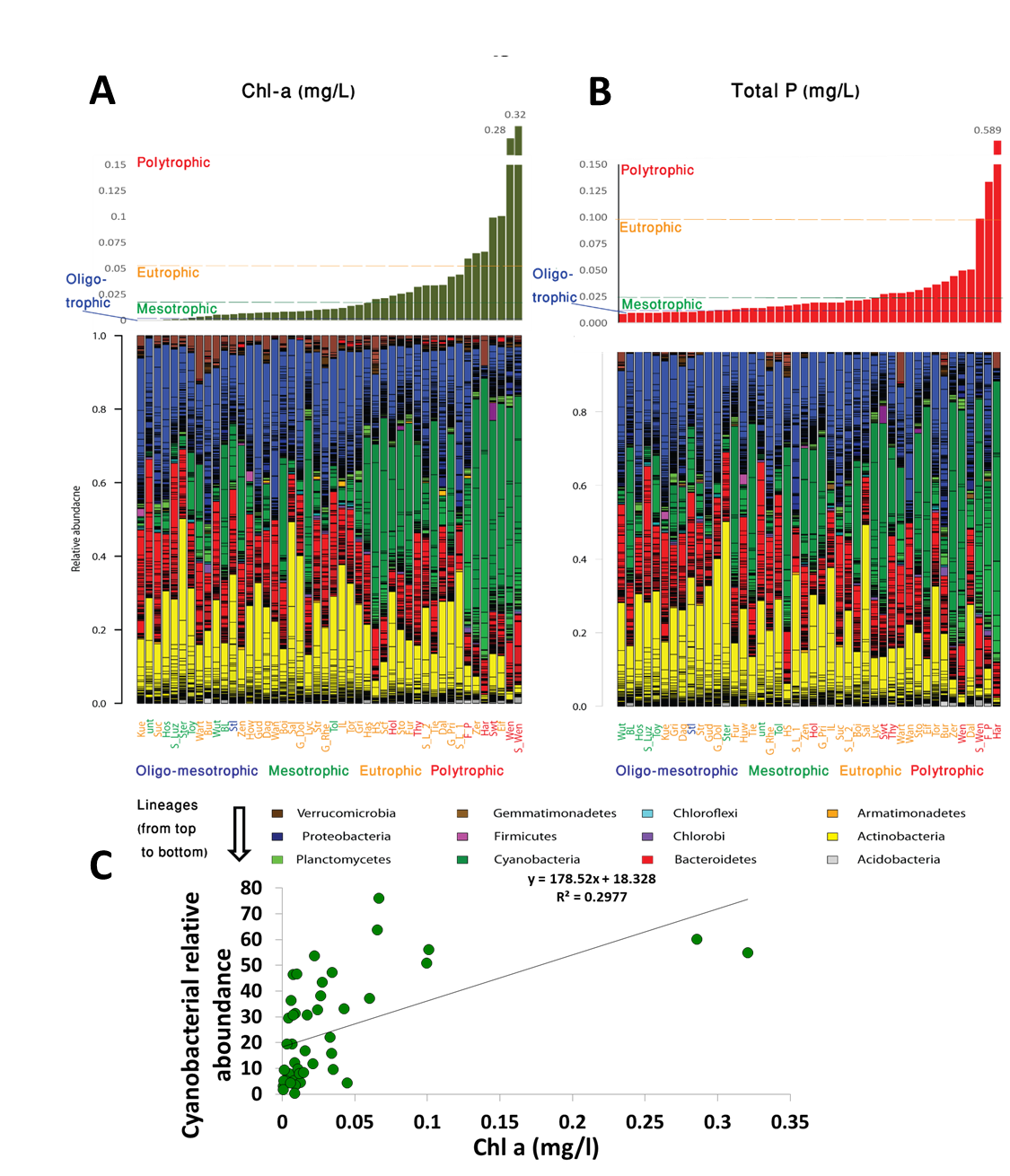
**

**Supplementary Figure 2. Relationship between trophic state and microbial community composition.** A, B) The population structure of the samples lakes, ordered by chlorophyll (a) and total P concentrations (B) at the time of sampling. The codes for the location sites are colored based on the trophic state shown in the supplementary excel file, which is based on the monitoring programs of the German federal states Mecklenburg-Vorpommern and Brandenburg, following in general the rules of the water framework directive of the European Union (see supplementary text for more information). A tendency is seen towards increased relative abundance of cyanobacteria when Chl a (A) and total P (B) are high. C) Correlation between chlorophyll concentration and relative abundance of cyanobacteria (Spearman R2=0.6, p<0.001). See also the NMDS of figure S3.

**
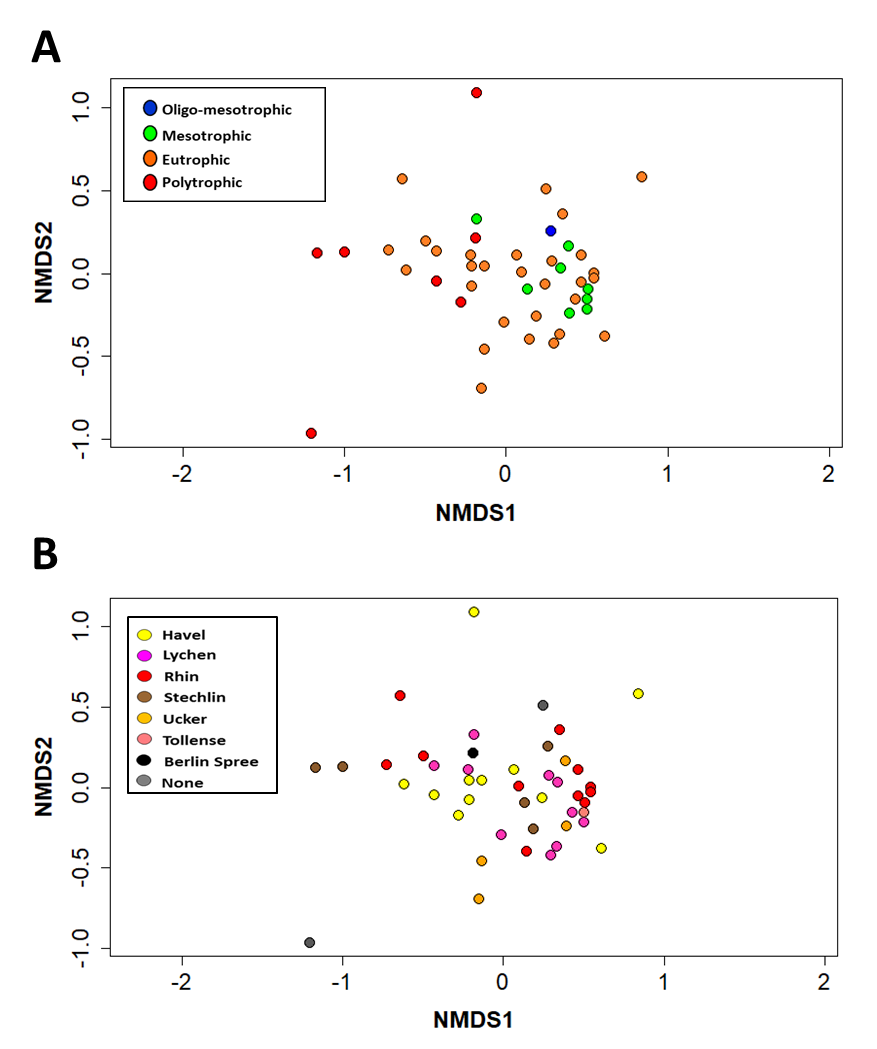
**

**
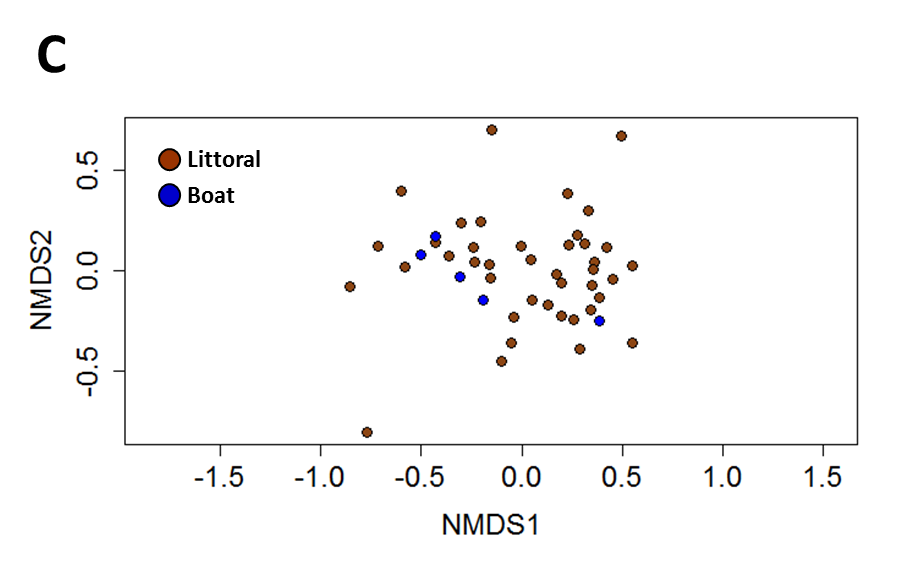
**

**Supplementary Figure 3.** NMDS analysis of the bacterial population structure, colored by trophic level (A), by drainage basin (B), and by whether the sampling was from the littoral or by boat (C). The NMDS was calculated using Bray-Curtis dissimilarities. K=2, Stress= 0.16

**
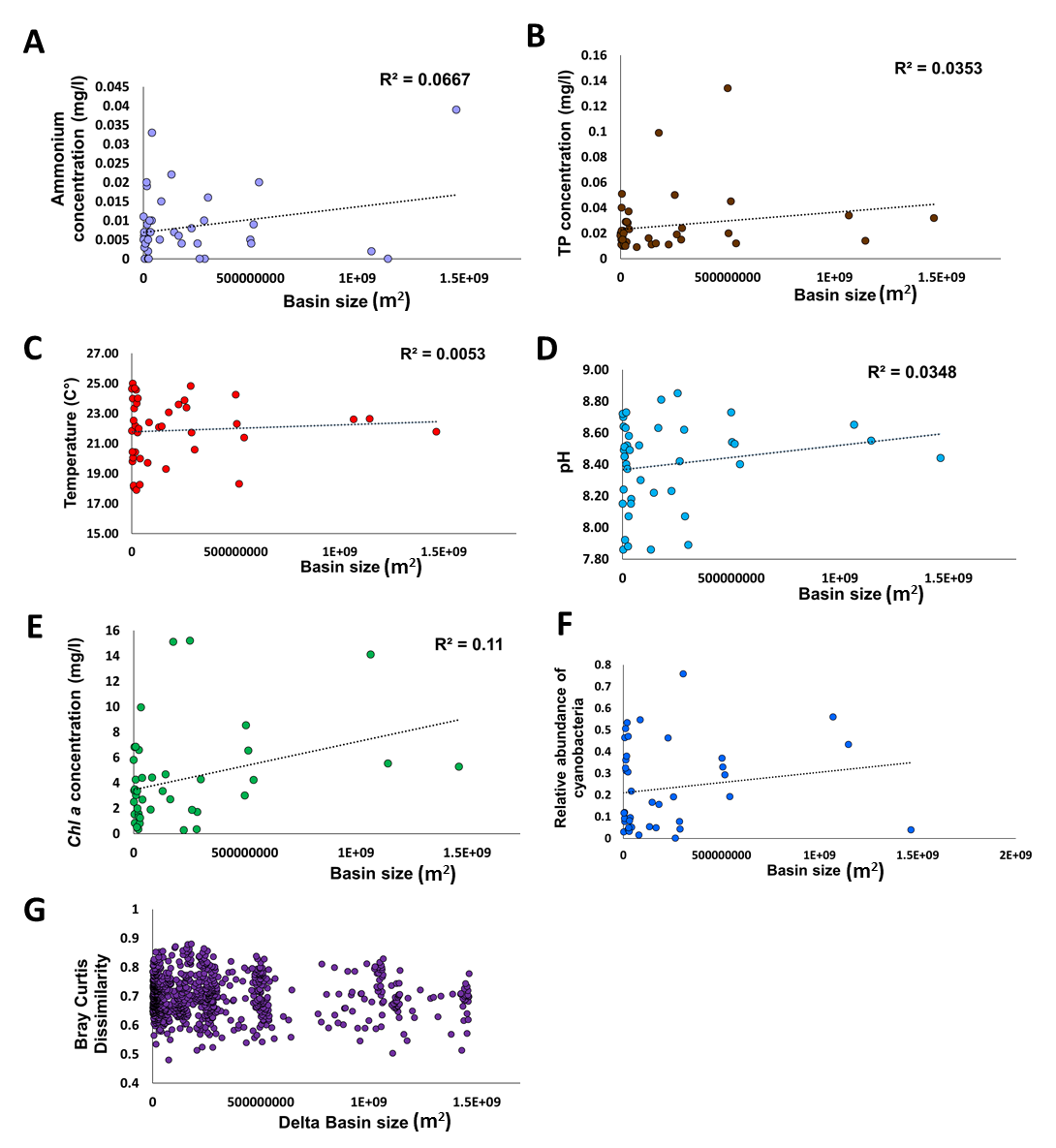
**

**Supplementary Figure 4. No clear correlation between basin size, water chemistry or microbiome composition. a-d) Drainage basin size vs ammonium (A), total phosphorus (B), temperature (C) and pH (D); no clear correlation can be seen. E) Low correlation between basin size and Chl a concentration, and F) No clear correlation between the fraction of cyanobacteria from the total microbiome and the basin size. G) No correlation between basin size and microbiome similarity. Each point represents the Bray-Curtis dissimilarity between two sampling points, plotted against the difference in size between the drainage basins of the two locations.**

**
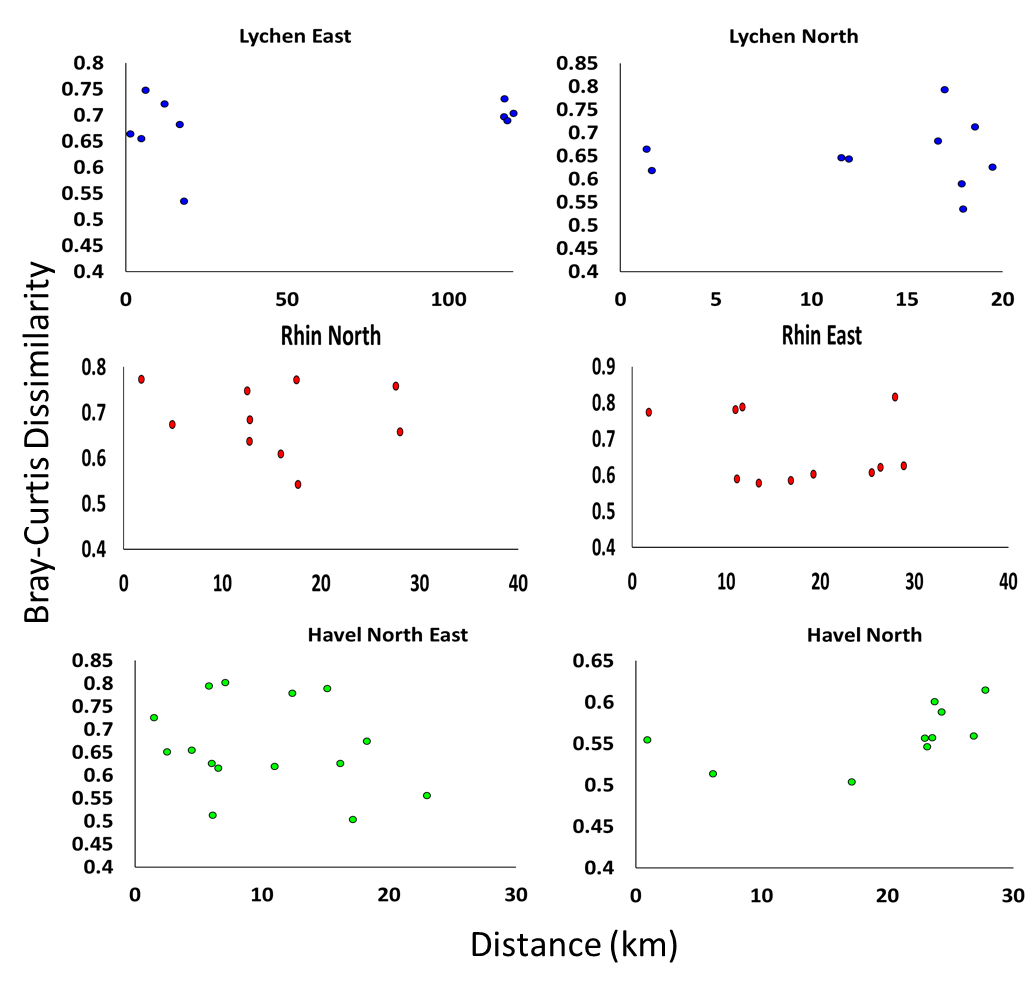
**

**Supplementary Figure 5. No distance decay patterns observed in sub-basins. The three large basins in the analysis were each divided into two smaller sub-basins, where all of the water bodies are directly connected. No distance-decay patterns were observed (Spearmans r ranged between 0.1 and 0.26, p values 0.3-0.7). The water bodies used for each sub-basin are as follows (see Supplementary excel File for water body codes). Lychen East: Lyc, Zen, Kue, Hos, Has; Lychen north: Lyc, Zen, HS, BL, Has; Rhin North: G.Rhe, Gri, Tie, Tor, Zer; Rhin East: Tor, Gud, G.Dol, Huw, Wut, Zer; Havel North-east: Thy, S.L_1, S.L_2, Sto, Fur, Swt; Havel North: G.Pri, Sto, EIF, Fur, .Swt**

**
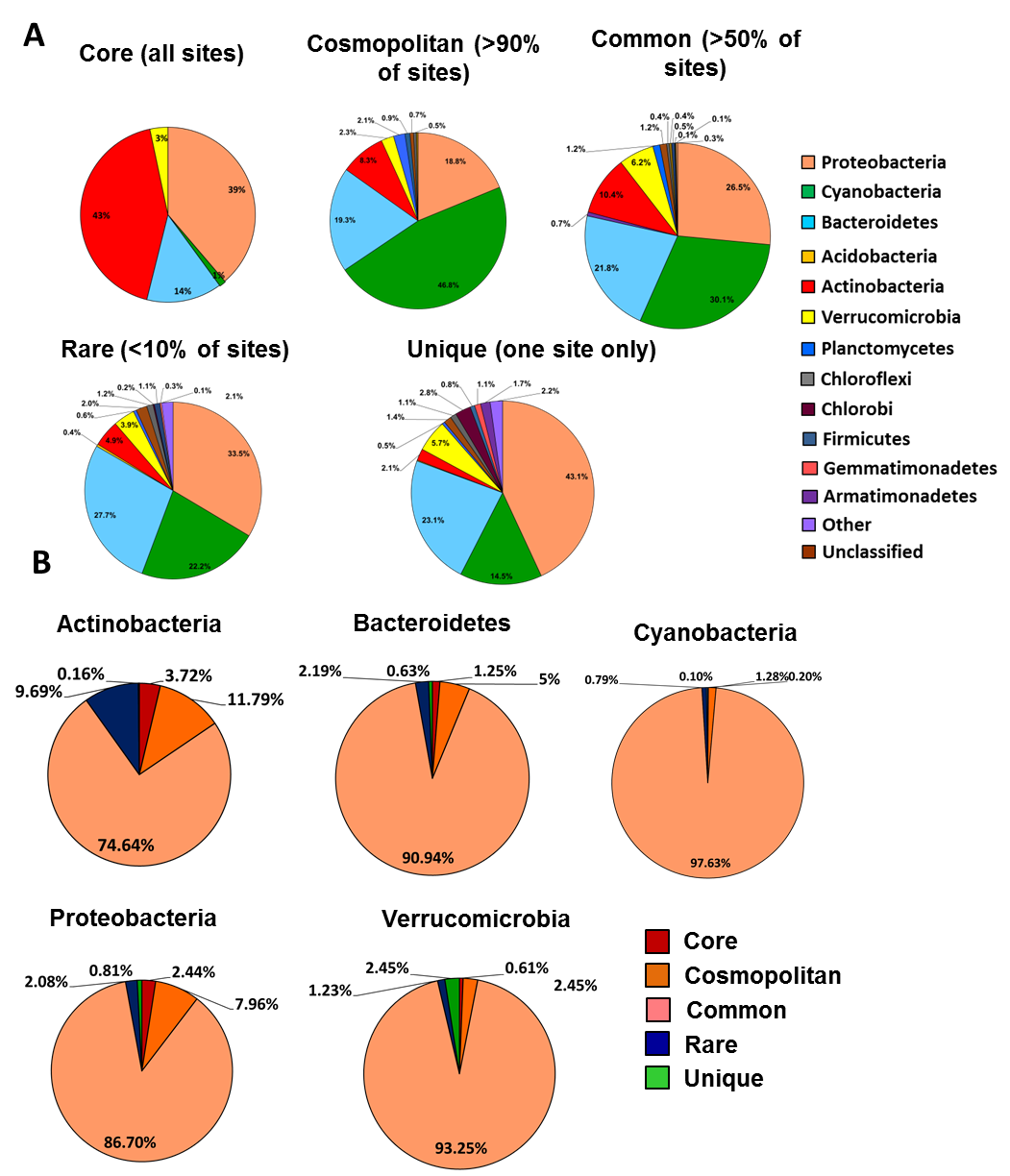
**

**Supplementary Figure 6. Relationships between different phyla and their relative abundance in the combined microbiome of all sampling sites. A) Relative abundance of bacterial phyla among core, cosmopolitan, common, rare and unique OTUs. The core OTUs, found in all sampling locations, are dominated by Proteobacteria and Actinobacteria. In contrast, few cyanobacterial OTUs are found in all sampling locations, whereas many are cosmopolitan (found in >90% of the locations). B) Distribution of core, cosmopolitan, common, rare and unique OTUs among the different phyla. Note the difference in the relative number of core and cosmopolitan OTUs between Actinobacteria, suggested to have similar communities in all sampling sites, and Cyanobacteria, which reveal niche differences between different orders.**

**
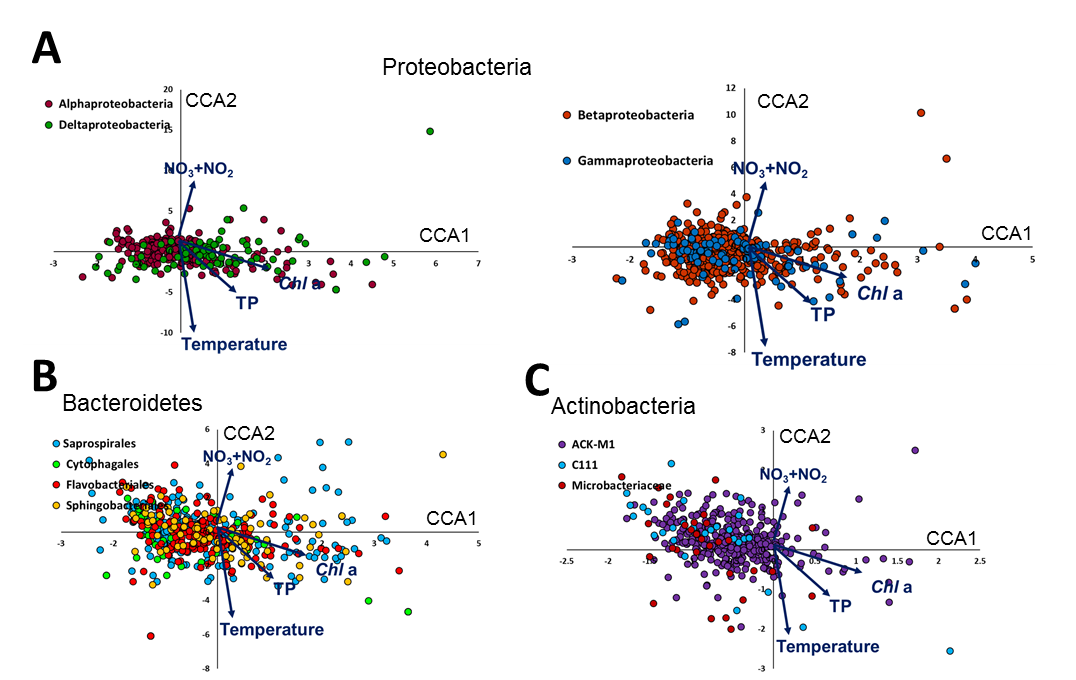
**

**Supplementary Figure 7. Absences of separation in the CCA ordination between several bacterial lineages: A) Proteobacteria orders, B) Bacteroidetes orders, C) Actinobacteria families. No clear differentiation is seen between the orders or families.**

**References**

Caporaso JG, Kuczynski J, Stombaugh J*, et al.* (2010) QIIME allows analysis of high-throughput community sequencing data. *Nature Methods* **7**: 335.

Langmead B & Salzberg SL (2012) Fast gapped-read alignment with Bowtie 2. *Nature Methods* **9**: 357.

Legendre P, Borcard D & Roberts DW (2012) Variation partitioning involving orthogonal spatial eigenfunction submodels. *Ecology* **93**: 1234-1240.

Oksanen J, Kindt R, Legendre P, O’Hara B, Stevens MHH, Oksanen MJ & Suggests M (2007) The vegan package. *Community ecology package* **10**: 631-637.

Salter, S.j., Cox, M.J., Turek, E.M., Calus, S.T., Cookson, W.O., Moffatt, M.F., Turner, P., Parkhill, J., Loman, N.J and Walker, A.W. (2014). Reagent and laboratory contamination can critically impact sequence-based microbiome analyses. BMC Bio 12, 87 doi:10.1186/s12915-014-0087-z

Schloss PD, Westcott SL, Ryabin T*, et al.* (2009) Introducing mothur: open-source, platform-independent, community-supported software for describing and comparing microbial communities. *Applied and Environmental Microbiology* **75**: 7537-7541.

Zhang J, Kobert K, Flouri T & Stamatakis A (2013) PEAR: a fast and accurate Illumina Paired-End reAd mergeR. *Bioinformatics* **30**: 614-620.
